# Differential depth distribution of microbial function and putative symbionts through sediment-hosted aquifers in the deep terrestrial subsurface

**DOI:** 10.1038/s41564-017-0098-y

**Published:** 2018-01-29

**Authors:** Alexander J. Probst, Bethany Ladd, Jessica K. Jarett, David E. Geller-McGrath, Christian M. K. Sieber, Joanne B. Emerson, Karthik Anantharaman, Brian C. Thomas, Rex R. Malmstrom, Michaela Stieglmeier, Andreas Klingl, Tanja Woyke, M. Cathryn Ryan, Jillian F. Banfield

**Affiliations:** 1 Department of Earth and Planetary Science, University of California, Berkeley, CA USA; 2 Department of Geoscience, University of Calgary, Calgary, AB Canada; 3 Department of Energy Joint Genome Institute, Walnut Creek, CA USA; 4 Plant Development and Electron Microscopy, Department of Biology I, Biocenter LMU Munich, Planegg-Martinsried, Germany; 5 Present Address: Group for Aquatic Microbial Ecology, Biofilm Center, Department of Chemistry, University of Duisburg-Essen, Essen, Germany; 6 Present Address: Department of Plant Pathology, University of California, Davis, Davis, CA USA

**Keywords:** Biogeochemistry, Environmental microbiology, Symbiosis, Metagenomics, Metagenomics

## Abstract

An enormous diversity of previously unknown bacteria and archaea has been discovered recently, yet their functional capacities and distributions in the terrestrial subsurface remain uncertain. Here, we continually sampled a CO_2_-driven geyser (Colorado Plateau, Utah, USA) over its 5-day eruption cycle to test the hypothesis that stratified, sandstone-hosted aquifers sampled over three phases of the eruption cycle have microbial communities that differ both in membership and function. Genome-resolved metagenomics, single-cell genomics and geochemical analyses confirmed this hypothesis and linked microorganisms to groundwater compositions from different depths. Autotrophic *Candidatus* “Altiarchaeum sp.” and phylogenetically deep-branching nanoarchaea dominate the deepest groundwater. A nanoarchaeon with limited metabolic capacity is inferred to be a potential symbiont of the *Ca*. “Altiarchaeum”. Candidate Phyla Radiation bacteria are also present in the deepest groundwater and they are relatively abundant in water from intermediate depths. During the recovery phase of the geyser, microaerophilic Fe- and S-oxidizers have high in situ genome replication rates. Autotrophic *Sulfurimonas* sustained by aerobic sulfide oxidation and with the capacity for N_2_ fixation dominate the shallow aquifer. Overall, 104 different phylum-level lineages are present in water from these subsurface environments, with uncultivated archaea and bacteria partitioned to the deeper subsurface.

## Abstract (web summary)

Analysis of a CO_2_-driven geyser over a complete eruption cycle showed temporal changes in microbial community composition and function, associated with eruption phase and aquifer water depth, and revealed a putative archaeal symbiosis.

## Main

Much remains to be learned about how microbial communities in the deep terrestrial subsurface vary with depth due to limited access to samples without contamination from drilling fluids or sampling equipment. Studies to date have analysed samples acquired by drilling^1–3^, from deep mines^4,5^, subsurface research laboratories^6,7^ and sites of groundwater discharge^8–11^. These investigations have shown that the terrestrial subsurface is populated by a vast array of previously undescribed archaea and bacteria. At one site, an aquifer in Colorado (Rifle, USA), the diversity spans much of the tree of life^12^ and includes organisms of the Candidate Phyla Radiation (CPR)^13^, which may comprise more than 50% of all bacterial diversity^14^, and many other previously undescribed bacterial lineages. Also present in the terrestrial subsurface are previously unknown or little known archaea, including members of the DPANN (Diapherotrites, Parvarchaeota, Aenigmarchaeota, Nanoarchaeota, Nanohaloarchaea)^11,15^, Altiarchaeum^10^, Lokiarchaeota^16^ and Aigarchaeota^17^.

A major question in subsurface microbiology relates to how organisms, and their capacities for carbon, nitrogen and sulfur cycling, vary along depth transects through the terrestrial subsurface. Some evidence pointing to taxonomic variation between 9 m and 52 m below the surface was obtained via a massive 16S ribosomal RNA gene survey at the Hanford Site^18^. Similar variation and change of two functional genes were also detected for two shallow aquifers that were accessed via drilling in Germany^19^. However, major groups of archaea and bacteria may have been overlooked due to sampling^13^ and primer bias^13, 20,21^ and the spatial variation in metabolic functions over depth transects including the deep subsurface (100 m below the ground) remains unexplored.

Crystal Geyser is a cold-water, CO_2_-driven geyser located geologically within the Paradox Basin, Colorado Plateau, Utah, USA^22^. Originally an abandoned oil exploration well, the 800-m deep vertical borehole has served as a geyser conduit whose regular and significant flow rate (since 1936) provides uncontaminated access to organisms present in underlying aquifers. Prior geological studies have defined the region’s hydrostratigraphy, including the transmissive Entrada, Navajo, Wingate and White Rim fractured sandstone aquifers (listed in order of increasing depth), which are separated by low-permeability confining units^23,24^, through which limited vertical connectivity for CO_2_, water and microbes is largely restricted to faults and fractures^25^. A nearby research borehole provided further geologic and aquifer geochemical information to 322 m below ground surface^26^. Time-series geochemical data collected over the ca. 5-day eruption cycle suggest that Crystal Geyser is primarily sourced from the Navajo Sandstone, with increased contributions from the shallower Entrada Sandstone during major eruptions, and increased fraction of deeper water during minor eruptions^26,27^.

A survey of ribosomal proteins predicted from metagenome sequences from Crystal Geyser microbial communities revealed the existence of a large phylogenetic diversity of previously unknown bacteria and archaea in this system^8^, and a genomic resolution study documented a high incidence of carbon-fixation pathways^9^. A remaining question relates to the source regions and distributions of these organisms. Here, we tracked the microbiology and the associated geyser discharge geochemistry continuously throughout the full 5-day geyser eruption cycle to test the hypothesis that groundwater from stratified aquifers sampled at different stages of the cycle has microbial communities that differ in both membership and function. Our analyses made use of a comprehensive collection of more than 1,000 newly reconstructed genomes, both from metagenomes and single cells, as well as detailed physical and chemical information that enabled linking of fluids to their groundwater source regions.

## Results

Continuous in situ (downhole) monitoring of the geyser water pressure throughout the field campaign defined the regular ~5-day period of the eruption cycle (Supplementary Fig. 1). Sampling was conducted over a complete eruption cycle (24–29 May, 2015) during which microbial cells were continuously collected onto 0.1 µm filters. Time series of downhole temperature, electrical conductivity, total dissolved gas pressure and water samples (for major ion, trace metal and dissolved gas analyses) were collected to associate specific microorganisms with water from different geyser eruption intervals and relative aquifer depths (Supplementary Fig. 1).

**Fig. 1:**
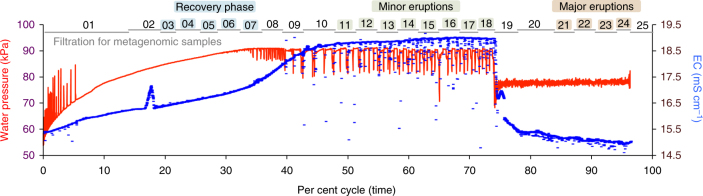
Crystal Geyser’s 5-day eruption cycle measured during the 2015 sampling period exhibited variations in downhole water pressure and electrical conductivity that define three phases. In each phase, electrical conductivity (EC) and geochemical measurements (6,710 measurements each, no technical replicates; Supplementary Fig. 1) are used to identify relative depths of source water compositions: intermediate for the recovery phase (2,330 measurements), deep for the minor eruptions (2,820 measurements) and shallow for the major eruptions (1,560 measurements). The numbered horizontal grey bars indicate the time periods for each metagenomic sample (25 samples in total) and coloured numbers indicate the grouping of samples from each phase.

Time series of water pressure, electrical conductivity and temperature showed three Crystal Geyser eruption phases previously observed^26,27^: the recovery (relatively low water level, no eruptions, light CO_2_ bubbling), minor eruptions (short eruptions of ~10 min every hour with elevated CO_2_ discharge) and major eruptions (constant eruption and heavy CO_2_ discharge^28^; Fig. 1). Average chloride concentrations ([Cl]) and baseline water temperature (16.9 °C; also observed in a year-long monitoring period; Supplementary Fig. 1d) indicate that, overall, the geyser water is primarily sourced from ~320 to 480 m depth, which mainly corresponds to the Navajo aquifer; Supplementary Fig. 1a–d). In minor eruptions, elevated electrical conductivity and [Cl] indicate increased contribution from deeper, more saline water (that is, possibly the Wingate aquifer or Paradox brine sourced from even greater depth; Supplementary Fig. 1c). In the major eruption phase, decreased electrical conductivity and [Cl], and elevated Ca, Sr and Fe concentrations, were consistent with an increased contribution from the shallower Entrada aquifer (Supplementary Fig. 1b). During the eruption-free recovery phase, in which the Crystal Geyser borehole slowly refilled after the major eruption phase, electrical conductivity gradually increased with the relative contribution of deeper groundwater up to (and during) the minor eruptions. During this time the water level increased ~3.5 m over 33.5 h, potentially enabling microbes to thrive in microaerophilic borehole-affected conditions. To simplify terminology, we henceforth refer to the source water compositions as relatively ‘deep’ during the minor eruptions, ‘intermediate’ (and borehole-affected) during the recovery phase and ‘shallow’ during the major eruptions. Similar phase variations in the relative depths of water composition were recently observed^27^.

Analysis of microbial community composition in the 2015 bulk samples made use of a relatively comprehensive database of genomic information for the Crystal Geyser system (see Supplementary Fig. 2 for sample processing and analysis overview). The genomic dataset included previously reported draft genomes from this system^9^ and new genomes reconstructed from size-fractionated samples obtained in April and October of 2014 (Supplementary Table 1). Samples included a post-0.2 µm fraction collected onto a 0.1-µm filter to enrich for community members with ultra-small cell sizes. Binning of assembled metagenomes from 27 different samples from five time points in 2014 used seven different algorithms (see Supplementary Methods) and resulted in 30,574 genomic bins (with multiple bins for the same genome generated by different algorithms). Selection of the best quality bin generated for any organism in each sample resulted in 5,795 bins for bacteria and archaea, of which 2,216 were considered to be at least medium quality (>70% completeness based on single-copy genes with less than three multiple single-copy genes). After curation based on guanine-cytosine content, coverage and taxonomy the database contains 1,215 genome sequences for 503 different archaeal and bacterial species (for details on genome numbers for each step, please see Supplementary Fig. 2; genome completeness is provided in Supplementary Table 2).

**Fig. 2:**
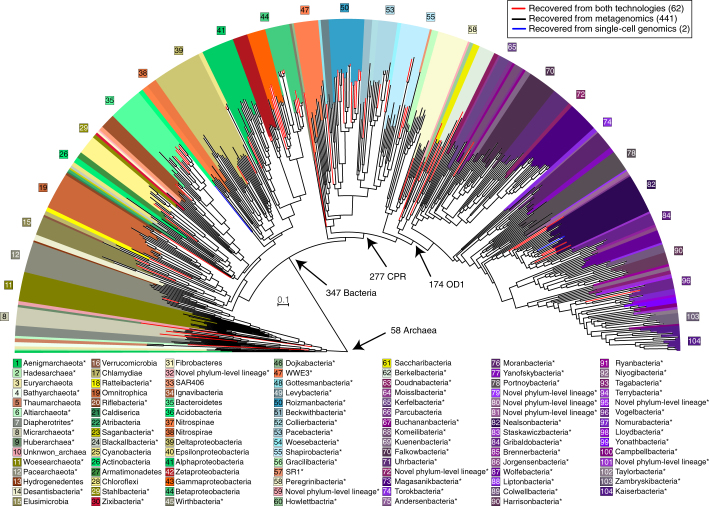
Diversity of recovered genomes based on 16 concatenated ribosomal proteins. Genomes were reconstructed for organisms from 104 different phylum-level lineages; 503 different lineages are shown (two lineages did not exhibit >50% alignment coverage and are thus not displayed). Phyla in bold were assigned names in this study. The scale corresponds to per cent average amino acid substitution over the alignment. Asterisks mark yet-to-be-cultivated phyla, which thus have a *Candidatus* status. OD1, Parcubacteria. A full tree with reference sequences can be found in Supplementary File 1.

To augment the genome-resolved metagenomics, we acquired 206 single amplified genomes (SAGs) from cells collected at one time point in the minor eruption and one time point during the recovery phase. SAGs were chosen for full sequencing and analysis if PCR-screening for 16S rRNA genes was positive, agnostic to the specific sequence. Only SAGs with assembly size of >100 kbp after multi-step contamination screening were considered further. This set comprised 183 SAGs, seven of which were of sufficient quality to be classified as medium-quality draft genomes ( > 70% complete, less than three multiple single-copy genes). We required alignments ≥98% nucleotide identity over >30% of the SAG to establish a match between SAGs and genomes from metagenomes. This approach was chosen because almost all of the SAGs were less complete than related draft genomes from metagenomes (Supplementary Fig. 3). In general, SAG sequences aligned well to the sequences of genomes from the metagenomes (Supplementary Fig. 3). We found that >70% of the SAGs (145, of which five were draft quality) were represented in the set of 503 draft-quality genomes from the metagenomes. Conversely, 63 of the 503 genomes from metagenomes were also detected by single-cell genomics. Two draft-quality SAG genomes were not binned from the metagenomes and thus were added to the database. One SAG is entirely absent in the metagenomes based on sequences of the ribosomal protein S3 and read mapping, and probably derived from a very rare organism. The 505 genomes in the database (Supplementary Table 2), which were derived via dereplication from a total set of 1,208 genomes (984 genomes from metagenomes, 222 genomes from a previous study^9^ and two single-cell genomes), represent archaeal and bacterial species that belong to 104 different phylum-level lineages (Fig. 2). Nine lineages were named as they were represented by least two genomes with significant phylogenetic distance to neighbouring phyla and thus may constitute previously unrecognized phylum-level lineages. In addition, six genomes may be from previously unknown phylum-level lineages but the lineages are currently only represented by a single genome. The majority of diversity was attributed to members of the CPR (Fig. 2).

**Fig. 3:**
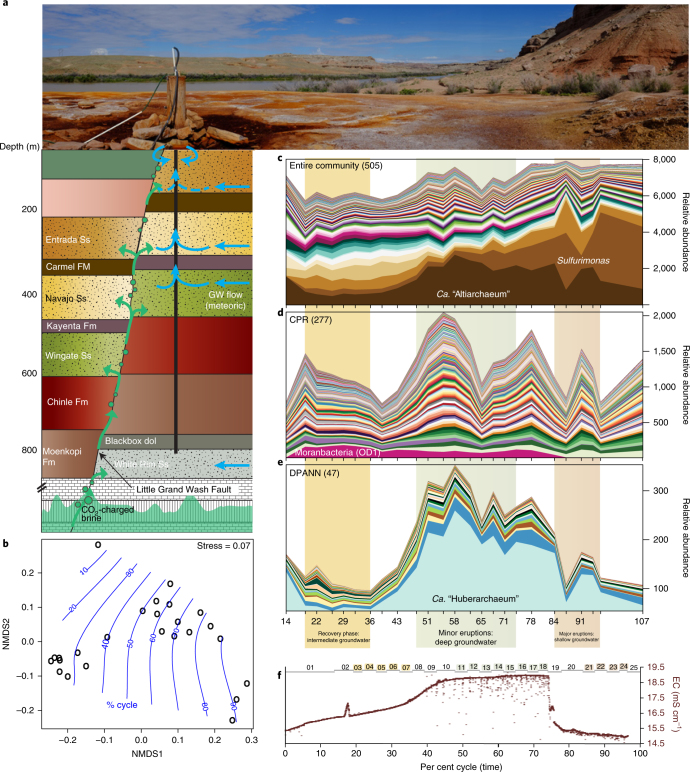
Hydrogeology and community composition of subsurface fluids sourced from Crystal Geyser throughout an entire eruption cycle. **a**, The Crystal Geyser site lies within one of the several natural CO_2_ reservoirs within the Paradox Basin. The CO_2_ was probably generated from thermal decomposition of Pennsylvanian-aged carbonate rocks^26, 51,52^. CO_2_ gas and brine formed by groundwater dissolution of Paradox evaporites migrate via faults and fractures^53,54^. **b**, The community profile of 505 organisms strongly followed the succession of the geyser eruptions (blue lines, NMDS). One data point corresponds to one metagenomic sample. The samples show a clear pattern following the succession of the geyser cycle. **c**, Entire community profile of 505 organisms tracked across the 5-day cycle of the geyser. Each colour corresponds to one genome. **d**,**e**, Profiles of the CPR and DPANN community, respectively, show an increase in the overall abundance during the minor eruptions when groundwater has the deepest source composition. **f**, Downhole electrical conductivity time series during the sampling of the cycle illustrating the individual phases of the geyser (6,710 samples were measured, see Fig. 1 and Supplementary Fig. 1). Number of biological replicates in panels **b**–**e** was 24. EC, electrical conductivity; GW, groundwater.

Mapping of metagenome reads to the set of 505 genomes showed that the genomes account for ~50% of the sequence data collected through the 2015 eruption cycle (Supplementary Table 4) and thus is representative of the community found in the Crystal Geyser ecosystem (Supplementary Fig. 4; morphological diversity of organisms is provided in Supplementary Fig. 5). Analysis of the community structure of the 25 different metagenomes using this approach revealed strong shifts in microbial composition over the cycle (Fig. 3b; Supplementary Table 5). The accuracy of relative abundance measures of individual genomes was confirmed for three species using quantitative digital droplet PCR (Supplementary Fig. 6). No physical or geochemical factor besides time, which corresponds to the source regions of the sampled water, could explain the observed changes in community composition (based on multivariate statistics; Supplementary Table 6). The community was dominated by species of the taxa *Candidatus* “Altiarchaeum”, *Sulfurimonas*, *Piscirickettsiaceae*, *Gallionellaceae* and Betaproteobacteria (in order of decreasing abundance, Fig. 3c). The set of CPR and archaea from the DPANN superphylum showed several peaks in relative abundance at several time points during the eruption cycle (Fig. 3d,e). Both groups had the highest cumulative abundance during the minor eruptions, when groundwater from the deepest source was sampled. The most abundant CPR (Moranbacteria^13^) was, however, prominent during the recovery phase (Fig. 3d). Overall, the cumulative abundances of DPANN and other archaea were significantly higher in the deep groundwater compared to shallow or intermediate (Supplementary Fig. 7).

**Fig. 4:**
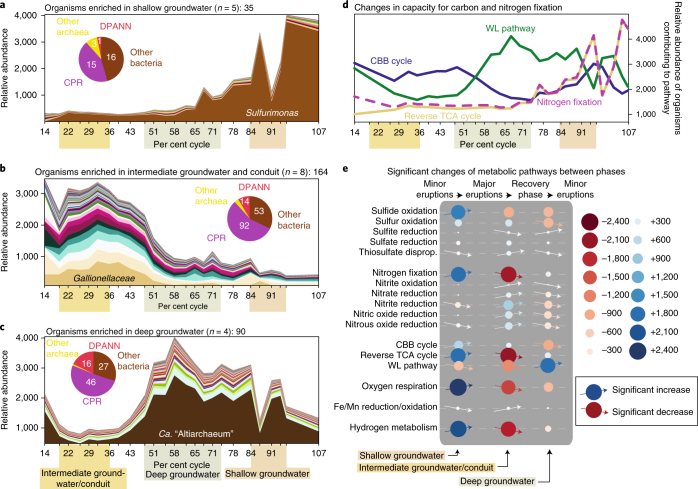
Microbial source tracking and changes of metabolic potential. **a**–**c**, The abundances of organisms that are significantly enriched in groundwater from the different depths (for details on organisms please see Supplementary Table 5, number of biological replicates are given in parenthesis of panels **a**–**c**). The pie charts indicate the diversity of CPR, DPANN, other bacteria, and other archaea associated with each relative depth. **d**, Different carbon fixation pathways predominate in groundwater from the three different depths. Nitrogen fixation and the reverse TCA cycle occur in one organism, *Sulfurimonas* (**a**). **e**, Metabolic pathway analysis shows distinct metabolic profiles associated with the groundwater from the different depths (individual metabolic capacities of each organism are listed in Supplementary Table 7). Each circle displays the cumulative relative abundance of genomes contributing to this single metabolic process. Arrows display if an increase or decrease is significant (*P* < 0.05). CBB, Calvin-Benson-Bassham; disprop., disproportionation.

When analysed one at a time, the majority of organisms (289, ~57%) were significantly enriched (false discovery rate-corrected *P* value <0.05) in one specific phase of the geyser and could thus be sourced to one of the groundwater depths (Fig. 4a–c). The shallowest groundwater was mainly populated by one *Sulfurimonas* sp. along with a few other bacteria and some archaea. Based on the genome sequence of *Sulfurimonas* sp., this organism was inferred to be a chemolithoautotroph, capable of nitrogen and carbon fixation as well as sulfide oxidation through oxygen respiration (Supplementary Table 7). The capacity for carbon fixation via the low-cost reductive TCA cycle (two ATP per pyruvate^29^) coupled to oxygen respiration may provide an ecological advantage for this species and is also indicative of microaerophilic conditions in the relatively shallow aquifer. In contrast to the shallow source, groundwater from intermediate depths had a great diversity of different organisms, the majority of which belonged to the CPR. The most abundant organism was a member of the *Gallionellaceae*, a family of bacteria well known for microaerophilic iron and sulfur oxidation at Crystal Geyser^8,9^. This organism also exhibited the highest genome replication rates of all bacteria in the study (average in situ replication rate (iRep) value of 2.5, maximum iRep value of 4.2; Supplementary Table 8), suggesting that it was also proliferating in the geyser conduit over the 33.5 h of the recovery phase. Its growth was probably favoured by microaerophilic conditions as well as sulfide and reduced iron in the geyser fluids. Potentially, other microorganisms enriched in this fraction may also have favoured the conditions in the borehole over the 33.5-hour recovery phase, during which the geyser had no water discharge. Consequently, the community sampled from the recovery phase represents the community from intermediate depths with distortions from microbial growth in the borehole. When deeper groundwater was discharged, the abundances of different DPANN archaea and *Ca*. “Altiarchaeum” were significantly increased. Diverse members of the CPR were still present in deep groundwater, although at low relative abundance.

The shallowest groundwater had a substantially higher capacity for microbial sulfide oxidation, nitrogen fixation and oxygen respiration, probably due to the presence of atmospheric gases. In contrast, the intermediate source and borehole community had the highest microbial capacity for reduction of various nitrogen compounds as well as thiosulfate disproportionation, metal reduction and oxidation. The deepest groundwater was enriched in several bacteria with the capacity for sulfite reduction, with carbon fixation mediated by the *Ca*. “Altiarchaeum”. The capacity for oxygen respiration decreased with increasing depth to the sourced groundwater.

Previously, we reported the operation of three carbon fixation pathways in bacteria and archaea from the Crystal Geyser communities, each of which requires substantially different amounts of energy^9^. While the Wood–Ljungdahl pathway requires approximately 1 mol of ATP for the generation of 1 mol pyruvate, the Calvin–Benson–Bassham cycle and the reverse TCA cycle require 7 mol and 2 mol, respectively^29^. Here, we show that the three carbon fixation pathways used by Crystal Geyser microorganisms were most abundant in different eruption phases, which reflect varying depths of source water composition (and borehole; Fig. 4e). The reductive TCA cycle was associated with a *Sulfurimonas* sp. that dominates the shallowest groundwater and also has the capacity for N_2_ fixation. The Calvin–Benson–Bassham cycle was enriched in bacteria associated with the intermediate groundwater as well as the borehole, and the Wood–Ljungdahl pathway was encoded in *Ca*. “Altiarchaeum” and Deltaproteobacteria genomes, and thus most highly represented in the deepest groundwater (Fig. 4d).

One previously undescribed archaeal phylum-level lineage within the DPANN branched next to Parvarchaeota (ARMAN-5) and *Nanoarchaeum equitans*. The 16S rRNA gene sequence of this species shared less than 67% identity with any 16S rRNA gene available in the SILVA database^30^ (and <78% with 16S rRNA gene fragments from environmental samples in the National Center for Biotechnology Information). We reconstructed 11 genomes for this species (including one from a single isolated cell) and estimated the genome size to be about 0.5 Mbp, which is similar to those of *Nanoarchaeum equitans* and some other DPANN^15,31^. We propose the name *Candidatus* “Huberarchaeum crystalense” (phylum *Ca*. “Huberarchaea”) for this archaeal lineage in honour of Prof. Robert Huber, pioneer in research on psychrophilic archaea and discoverer of *Ca*. “Altiarchaeum”.

Although enzymes for modification of purine and pyrimidine bases were encoded in the genome of *Ca*. “H. crystalense” (for example, via methylated folate), it is predicted to be incapable of de novo nucleotide synthesis (Supplementary File 2). The genome encodes a near-complete set of aminoacyl transfer RNA synthetases, proteins for replication and repair of DNA and translation and transcription machinery. Amino acids, whose biosynthesis pathways were lacking, are probably acquired via five different proteases. It has enzymes for glycosylating proteins and lipids and a near-complete pathway for lipid biosynthesis, observations that support the claim that this is a cellular organism. Protein export was probably accomplished via an encoded sec-pathway. Based on the limited metabolism of *Ca*. “H. crystalense”, we infer a symbiotic lifestyle. Interaction of the symbiont and a host may be mediated via large surface proteins, some of which are Cys-rich^32^. One of the extracellular, membrane-anchored Cys-rich proteins is predicted to bind calcium (Supplementary Fig. 8)^33^, a function also commonly found in hemolysin proteins. Hemolysin proteins destroy cell membranes, an activity that might be pivotal for *Ca*. “H. crystalense” to access metabolites from its host.

**Fig. 5:**
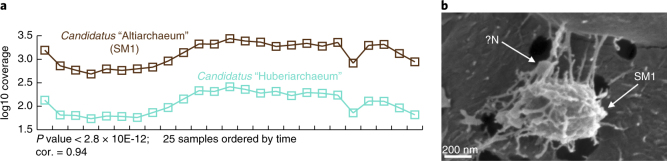
Putative symbiotic interaction of *Ca*. “Altiarchaeum” and *Ca*. “H. crystalense” **a**, Linear correlation analysis of relative abundance of the two archaea across 25 metagenome samples (full cycle of the geyser). **b**, Scanning electron micrograph of what are inferred to be *Ca*. “Altiarchaeum” cells (“SM1”) taken during the minor eruptions of the geyser. Tiny cell-like structures appear to be attached to the surface (“?N”). This structure was observed in two out of five samples taken for scanning electron microscopy analysis from the geyser fluids. cor., correlation coefficient. More images are available under Supplementary Fig. 9.

Within the whole geyser community, *Ca*. “H. crystalense” is the seventh most abundant organism. Notably, its abundance correlated significantly with that of the dominant organism, *Ca*. “Altiarchaeum” (linear correlation, *P* value < 2.8 × 10E-12; Fig. 5a). Based on the correlation of abundance patterns, we suggest that *Ca*. “H. crystalense” is a symbiont of the *Ca*. “Altiarchaeum”. Some support for this may be provided by scanning electron microscope images, which showed small rounded structures of approximately 0.15-µm diameter attached to larger cells (Fig. 5b, Supplementary Fig. 9). We infer that the larger cells are *Ca*. “Altiarchaeum”, based on the distinct hami-like appendages^10^, and that *Ca*. “H. crystalense” are episymbionts. Interestingly, both genomes exhibited very high levels of fragmentation, an indication of high levels of strain heterogeneity within both populations. The diversification of the *Ca*. “Altiarchaeum” host in its deep subsurface habitat might drive coevolution of *Ca*. “H. crystalense”. The shared characteristic of strain heterogeneity may also support the inference of their interaction.

## Discussion

Our microbiological investigation clearly demonstrated a strong stratification of microbial community composition and microbial function with relative groundwater source depths. Groundwater sampled from all three relative depths was dominated by autotrophs. The main pathway used for carbon fixation in the deeper subsurface is the one with the lowest energy cost, the Wood–Ljungdahl pathway, possibly because the deep biosphere is the most energy limited. Use of this pathway for provision of organic carbon was reported recently for other deep biosphere communities^3,6^. This pathway is also central to metabolism of methanogens, archaeal autotrophs found in the deep subsurface^34^. Reliance on the Wood–Ljungdahl pathway for CO_2_ fixation may be a widespread phenomenon in such environments. Our results indicate that the carbon provided by primary producers operating different carbon fixation pathways sustains a wide variety of bacteria and archaea in the subsurface.

**Fig. 6:**
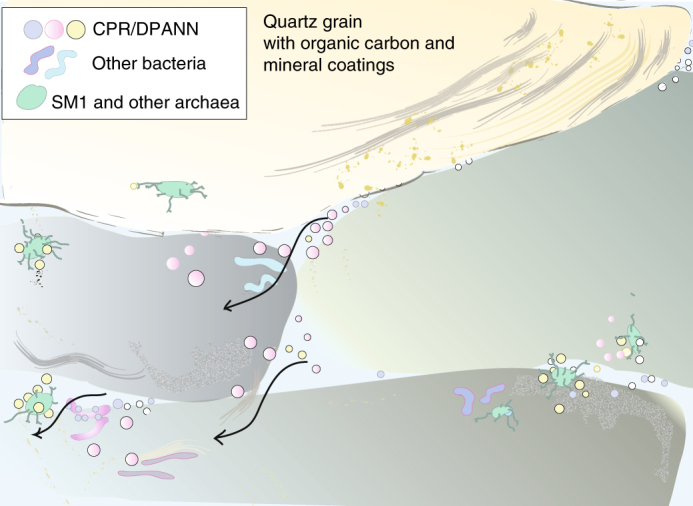
Conceptual representation of a relatively stable microbiome in deeper sandstone aquifer sources. The microbiome is dominated by *Ca*. “Altiarchaeum” (SM1) and their putative DPANN symbionts and populated by many CPR and other bacteria, some of which are probably symbiotic partners for CPR. We envision facile distribution of the very small CPR and DPANN cells through the sandstone pore spaces, providing periodic opportunities for establishment of the symbiont–host interactions that are probably required for CPR and DPANN cell replication. This figure provides a conceptual diagram of generalized microbial habitats in the aquifer based on an approximate pore size of sandstone. However, we note the subsurface is a heterogeneous three-dimensional system and physical properties will vary substantially^55^. The Carmel and Kayenta formations are expected to act as aquitards (confining barriers) that separate the high permeability sandstone aquifers (Fig. 3a), with each aquifer largely confined, both hydrologically and microbiologically, from other aquifers by these low-permeability shale/mudstone units^56^. This physical separation by low-permeability units probably contributes to the distinctive microbial communities associated with the three relative groundwater source depths as documented in the study.

This study adds to a growing body of literature that suggests that terrestrial subsurface regions are biodiversity hotspots^6,12^. Notably, we find that deeper regions can be particularly enriched in candidate phyla bacteria (especially CPR), DPANN archaea and other deep-branching archaea. The CPR were the most diverse organisms in the system. Intriguingly, many of these enigmatic CPR and DPANN are inferred to be symbionts^13,15^, probably episymbionts of other bacteria or archaea^35^. One highly abundant DPANN is a putative episymbiont of the most abundant archaeon, *Ca*. “Altiarchaeum”; however, further investigations are necessary to confirm this association. The putative symbiotic relationship between *Ca*. “Altiarchaeum” and *Ca*. “H. crystalense” could be analogous to that described between *Ignicoccus hospitalis* and *Nanoarchaeum equitans*^36^. Although *Ca*. “Altiarchaeum” is found elsewhere in the subsurface^10^, *Ca*. “H. crystalense” has not been detected in other metagenomic studies. The frequent detection of CPR and DPANN in groundwater, as found in this and other studies^6, 8, 9, 12, 13, 15^, may reflect the advantage of existence as ultra-small cells that can be readily distributed through sediment pore spaces, allowing periodic encounters with potential host organisms (Fig. 6). Highly interdependent lifestyles and intimate metabolic connections among community members may be an adaptation to constant low-nutrient conditions at depth.

## Methods

### Water chemistry and isotopes

Downhole electrical conductivity, water pressure and temperature were monitored using a Solinst LTC logger located in Crystal Geyser borehole about 8.5 m below ground surface. Water samples for major ions and trace metals were collected hourly at a pumping rate of 0.2 l min^-1^ over the eruption cycle from the borehole from a sampling tube inserted to 8.5 m below ground surface, and from the Green River. During two individual minor eruptions, samples were collected approximately every 10 min. Samples were field filtered to 0.2 μm by hand using Acrodisc syringe filters and a plastic syringe before collection into prerinsed 60 ml scintillation vials and then acidified to pH 2 with high-purity nitric acid for sample preservation^37^. The bottles were frozen for transport to the University of Calgary. Alkalinity was measured in the laboratory using an Orion Autochemistry 960 Autotitrator with 0.2 N sulfuric acid within 1 month of collection and expressed as HCO_3_ concentration. Major element and trace metal concentrations were determined using Inductively Coupled Plasma Emission Spectrometry and Inductively Coupled Plasma Mass Spectrometry, respectively^38^, at the Geologic Survey of Canada in Calgary.

### Dissolved gas collection and analysis

Water samples for dissolved gas composition were taken simultaneously with water chemistry and isotope samples, collected using the inverted bottle method^39^ in 12 ml glass bottles capped with precision seal silicone septa caps. All samples were refrigerated until analysis at the University of Calgary. Dissolved gas compositions were determined by gas chromatography using headspace extraction. Due to our primary interest in CO_2_ gas in this system, we used a headspace to sample water ratio of 3:1 and shaking time of 12 min at 400 rpm^40^. Headspace samples were injected onto an HP 5890 (Hewlett-Packard) gas chromatograph with a Hamilton gas-tight syringe via a six-port, two-position sampling valve. The gas chromatograph was outfitted with parallel Rt-Msieve 5 A (Restek, 30 m × 0.32 mm) and Rt-Q-PLOT (Restek, 30 m × 0.53 mm), and data were collected using an HP 3396 Series II Integrator (Hewlett-Packard).

### Genome-resolved metagenomics and single-cell genomics

Methods for genomic analysis of the 2014 datasets (including estimation of genome completeness) can be found in the Supplementary Methods.

### Crystal geyser genome database

The genome database was constructed from genomes, from metagenomes and from single-cell genomes (SAGs) collected in 2014. First, all curated, newly binned genomes from metagenomes (985 in total) were combined with 222 previously published genomes^9^ and clustered based on 98% nucleotide identity. One representative of each genome cluster was chosen based on the highest completeness (single-copy genes) and lowest amount of contamination (multiple single-copy genes) following the formula: score = single-copy genes—2x multiple single-copy genes^9,12^. In cases of ties, the genome with the highest N50 was chosen. The resulting 503 archaea and bacteria were then compared against draft-quality SAGs (at least 70% complete) using 98% nucleotide identity. Two draft-quality SAGs were not covered by the genomes from metagenomes and were thus added to the Crystal Geyser database that consists of 505 archaeal and bacterial species used for downstream analyses. A schematic overview of the procedure is presented in Supplementary Fig. 2.

### Comparison of genomes from metagenomes to SAGs

Whole-genome alignment of genomes from metagenomes^9^ was performed at 98% nucleotide identity. If a SAG shared more than 30% of its genomic content with a genome from a metagenome (which were at least 70% complete), the SAG was considered to be represented by the genome from the metagenome (Supplementary Fig. 4).

### Phylogeny of bacteria and archaea

Phylogenetic placements of the 505 archaea and bacteria in the Crystal Geyser database were determined from a tree computed from 16 ribosomal proteins^14^ and included 3,609 sequences (including reference sets from previous studies^12,14^). Bacterial ribosomal proteins were extracted using usearch^41^ against a public database^9^ (https://github.com/AJProbst/sngl_cp_gn), while archaeal ribosomal proteins were first selected by searching against Hidden Markov Models (HMMs)^42^ built from a previous dataset^14^ (to exclude A/E type) and then annotated against UniRef^43^. Individually aligned protein sequences^44^ were end trimmed and gaps (<5% coverage) were removed before concatenation of protein sequences. Only sequences of genomes that spanned at least 50% of the alignment were included in the phylogenetic analysis; others were classified using ribosomal protein S3 or 16S rRNA gene sequences. Trees were computed as described earlier^14^. Taxa from Probst et al., 2016 that were redefined in their taxonomy are listed in Supplementary Table 2.

### Tracking community members across time

In 2015, time resolution of the Crystal Geyser community was achieved by near-continuous filtration of groundwater onto 0.1-µm filters (ZTECG, Graver Technologies, Glasgow, USA) that we recovered at 25 different time points over a time course of nearly 5 days (114 h). Filtration was performed for an average of 4.6 h per sample and sampling spanned one entire eruption cycle of the geyser (Fig. 1). Metagenomic DNA was extracted from the samples^8^ and paired-end sequenced (Illumina HiSeq 2500, Supplementary Table 1). Reads of 150 bp were quality filtered (see Supplementary Methods) and mapped onto the de-replicated genome set of 505 organisms using bowtie2^45^ (default settings), allowing three mismatches per 150-bp read (98% identity)^46^. Read coverage was normalized by genome size and relative abundances of each genome in each sample were normalized by number of reads per sample using the equation *A*_r_ = *N*_m_/*N*_s_ * (*r* * *l*) *g*^-1^, where *A*_r_ is the relative abundance of the genome in a particular sample, *N*_m_ is the maximum number of reads of all metagenome samples, *N*_s_ is the total number of reads of that particular sample, *r* is the number of reads of that particular sample that mapped to the genome, *l* is the average read length and *g* is the length of the genome.

iRep values were calculated with one mismatch per read as described earlier^46^. Relative abundance measure using metagenomics was confirmed using quantitative digital droplet PCR (ddPCR), for which the method can be found in the Supplementary Methods.

### Microbial metabolism from genomics

Microbial metabolism from genomics was predicted as described earlier^9,12^. In brief, genes for each genome were predicted using prodigal^47^ with the respective genetic code and key metabolic genes for various sulfur, nitrogen, hydrogen and metal redox processes were predicted using HMMs^12,42^. In addition, functional predictions against KEGG were performed on the basis of HMMs including hits with e-values < E-10^9^. As such, the genetic potential of organisms for carbon fixation and oxygen respiration was based on the presence of all key enzymes and a pathway coverage of at least 60% in the KEGG module.

### Microbial community statistics

Ordination analyses of microbial community structure was performed using a Bray–Curtis distance measure and non-metric multidimensional scaling (NMDS) in the R programming environment^48^. Influence of environmental factors as provided in Supplementary Table 1 were determined by BioENV (Bray–Curtis dissimilarity, Spearman correlation) and plotted onto the NMDS^49^. Microbial source tracking of organisms and changes in microbial metabolism between different groundwater source depths based on cumulative abundance of organisms was performed using analysis of variance coupled to a Tukey honest significant difference post hoc test. Sample designations of the different depths correspond to those provided in Fig. 1. All *P* values that were affected by multiple testing were corrected for false discovery rate using the Benjamini–Hochberg procedure^50^.

### Scanning electron microscopy

Methods can be found in the supplementary documents.

### Life Sciences Reporting Summary

Further information on experimental design is available in the Life Sciences Reporting Summary.

### Data availability

SRA accession numbers for metagenomes of each sample are provided in Supplementary Table 1. All genomes from metagenomes included in this study were deposited at NCBI under Bioprojects PRJNA362739, PRJNA349044 and PRJNA297582. Genomes from metagenomes and single-cell genomes are also available under: http://ggkbase.berkeley.edu/CG_2014_505_non-redundant_genomes/organisms, http://ggkbase.berkeley.edu/CG_2014_genomes_from_metagenomes/organisms and http://ggkbase.berkeley.edu/CG_2014_SAGs/organisms.

## Supplementary information

Supplementary Information Supplementary Methods, Supplementary Figures 1–9, Supplementary Table 4 and Supplementary References.

Life Sciences Reporting Summary

Supplementary File 1 Phylogenetic tree based on 16 concatenated ribosomal proteins.

Supplementary File 2 HMM profile of all 11 genomes of *Candidatus* ’Huberarchaeum crystalense’. The completeness of each pathway is displayed and the individual enzymes for each KEGG module are displayed by clicking onto the respective numbers (counts) of each module. The displayed predictions were retrieved via HMM search against each single KEGG enzyme with e-values < E-10 as described in the methods. For details on HMM generation please see Probst et al., 2016.

Supplementary Table 1 Sample overview. All samples had a read length, or were trimmed to a read length, of 150 bp.

Supplementary Table 2 Genome completeness of 983 genomes from metagenomes and 183 single-cell genomes based on 51 bacterial single-copy genes and 38 archaeal single-copy genes.

Supplementary Table 3 Overview of the taxonomy of the 505 genomes and novel phylum names that have *Candidatus* status.

Supplementary Table 5 Normalized relative abundance of organisms from Crystal Geyser across the 25 metagenome samples (see Supplementary Table 3). Relative abundance values are based on bowtie229 mapping allowing a maximum of three mismatches per read (98% identity; see methods). Column B, C and D indicate where the respective organism was enriched.

Supplementary Table 6 Overview of the environmental variables collected for each metagenomic sample. Continuously collected data for variables like temperature were averaged over the sampling time, in which the metagenomic sample was acquired. BioENV was performed on all continuous variables listed, except sampling date.

Supplementary Table 7 Metabolic profile of 505 species detected at Crystal Geyser based on recovered genomic content.

Supplementary Table 8 iRep33 values of organisms across the different metagenomes of the geyser cycle. iRep analysis is based on mapping reads using bowtie229 allowing one mismatch per read.
