# Supplementary material for: Differential depth distribution of microbial function and putative symbionts through sediment-hosted aquifers in the deep terrestrial subsurface
Source: Nat Microbiol. 2018 Jan 29;3(3):328–36. doi: 10.1038/s41564-017-0098-y (PMC6792436; doi:10.1038/s41564-017-0098-y)
Supplement: Supplementary file 1 — Supplementary Methods, Supplementary Figures 1–9, Supplementary Table 4 and Supplementary References. [file 41564_2017_98_MOESM1_ESM.pdf]

nature microbiology

SUPPLEMENTARY INFORMATION
https://doi.org/10.1038/s41564-017-0098-y

In the format provided by the authors and unedited.

Differential depth distribution of microbial function and putative symbionts through sediment-hosted aquifers in the deep terrestrial subsurface

Alexander J. Probst[1,5,7], Bethany Ladd[2,7], Jessica K. Jarett[3], David E. Geller-McGrath[1], Christian M. K. Sieber[1,3], Joanne B. Emerson[1,6], Karthik Anantharaman[1], Brian C. Thomas[1], Rex R. Malmstrom[3], Michaela Stieglmeier[4], Andreas Klingl[4], Tanja Woyke[3], M. Cathryn Ryan[2*] and Jillian F. Banfield[1*]

[1]Department of Earth and Planetary Science, University of California, Berkeley, CA, USA. [2]Department of Geoscience, University of Calgary, Calgary, AB, Canada. [3]Department of Energy Joint Genome Institute, Walnut Creek, CA, USA. [4]Plant Development and Electron Microscopy, Department of Biology I, Biocenter LMU Munich, Planegg-Martinsried, Germany. Present address: [5]Present address: Group for Aquatic Microbial Ecology, Biofilm Center, Department of Chemistry, University of Duisburg-Essen, Essen, Germany. [6]Present address: Department of Plant Pathology, University of California, Davis, Davis, CA, USA. [7]These authors contributed equally: Alexander J. Probst and Bethany Ladd. *e-mail: cryan@ucalgary.ca; jbanfield@berkeley.edu

© 2018 Macmillan Publishers Limited, part of Springer Nature. All rights reserved.

Supplementary Information for

Differential depth-based distribution of microbial function and putative symbionts through sediment-hosted aquifers in the deep terrestrial subsurface

Alexander J. Probst[1,#,@], Bethany Ladd[2,#], Jessica K. Jarett[3], David E. Geller-McGrath[1], Christian M.K. Sieber[1,3], Joanne B. Emerson[1,⊙], Karthik Anantharaman[1], Brian C. Thomas[1], Rex R. Malmstrom[3], Michaela Stieglmeier[4], Andreas Klingl[4], Tanja Woyke[3], M. Cathryn Ryan[2,*] and Jillian F. Banfield[1,*]

[1]Department of Earth and Planetary Science, University of California, Berkeley, CA 94720, USA
[2]Department of Geoscience, University of Calgary, Calgary, AB, T2N 1N4, Canada
[3]Department of Energy Joint Genome Institute, Walnut Creek, CA 94598, USA
[4]Plant Development and Electron Microscopy, Department of Biology I, Biocenter LMU Munich, 82152 Planegg-Martinsried, Germany

[#]Authors contributed equally to this work

* Corresponding authors: jbanfield@berkeley.edu, cryan@ucalgary.ca

Table of contents:

1. **Supplementary Methods**
2. **Supplementary Figures**
3. **Supplementary Tables**
4. **Supplementary Files**
5. **References**

1. Supplementary Methods

Additional information on borehole completion, water sampling, pumping and geyser discharge flow rates. Relatively little information exists about the drilling and construction of the 'Glen Ruby' borehole that formed the Crystal Geyser. Drilled in 1935-1936, a sparse drill log is available from the State of Utah, but no official well construction records have been found to the best of our knowledge. Multiple sources state that the steel surface casing reaches ~30 m below ground[1–3], and 1936 news reports in the Times Independent Newspaper suggested the well was cased with 25.4 cm diameter casing to ~182 m, and 15.24 cm casing to at least ~585 m[1]. Since the borehole was blocked with rubble at about 10 m depth, there exists no deeper monitoring information related to where CO_2 and water enter the borehole.
The pumping rate for hourly geochemical sampling was (~0.2 l/min for ~5 minutes each hour, or ~ one litre per hour) and constant pumping for microbial sampling occurred an average rate of 4 l/min. The recovery period was characterized by a gradual increase in water level (~3 m over 1.5 days) in the well, with no surface water discharge. Visual observation made it apparent that it is unlikely that the overall sampling rate (~4.2 l/min) was significant compared to natural geyser flushing rate during the minor and

[@] current address: Group for Aquatic Microbial Ecology (GAME), Biofilm Center, Department of Chemistry, University of Duisburg-Essen, 45141 Essen, Germany
[⊙] current address: Department of Plant Pathology, University of California, Davis, Davis, CA 95616

major eruption periods (visually estimated to be >400 l/min during eruption). The surface pool around Crystal Geyser was observed to fill during each individual minor eruption, a trend that became progressively stronger and more frequent over the minor eruption period. Intermittent overland flow was observed towards the end of the minor eruption period. Maximum water discharge from Crystal Geyser, with significant overland flow to the Green River, was observed during the major eruption period.

Although overland flow rates were not gauged in this study, discharge during Crystal Geyser's eruption was previously estimated at ~1000 l/min[2]. This number is more than three orders of magnitude greater than our sampling rates, showing a sampling volume to flushing volume ratio of ~.0042.

Genome-resolved metagenomics of size-fractionated samples. Microbial size filtration from Crystal Geyser fluids was performed using two different sampling systems. One system involved sequential filtration of aquifer fluids on 3.0-µm, 0.8-µm, 0.2-µm, and 0.1-µm filters (polyethersulfone, Pall 561 Corporation, NY, USA) followed by freezing on dry ice as described earlier[4]. The second system was designed to filter high volumes of water sequentially onto 2.5-µm, 0.65-µm, 0.2-µm and 0.1-µm filters (ZTECG, Graver Technologies, Glasgow, USA). After collection, filters were reverse flushed onto smaller filters (Pall 561 Corporation, NY, USA, see above) and both filters were immediately frozen on dry ice for processing in the laboratory.

Metagenomic DNA was extracted from the filters as described earlier using MoBio PowerMax soil kit[4]. DNA was subjected to paired-end illumina HiSeq sequencing at the Joint Genome Institute (Supplementary Table 1). Obtained reads were hard trimmed to 150 bps and quality-filtered using BBduck (https://sourceforge.net/projects/bbmap/) and Sickle (https://github.com/najoshi/sickle). Assembly of high-quality reads was performed using IDBA_UD[5] with standard parameters and genes of assembled scaffolds (>1kb) were predicted using prodigal (-m -p meta). 16S rRNA and tRNAs genes were searched for using CMsearch[6] and tRNAscan-SE[7], respectively.

It has previously been shown that the usage of multiple binning algorithms outperforms the usage of one single algorithm, even if that one might outperform the others in a pairwise comparison[8]. Consequently, we binned genomes from metagenomes using seven different binning algorithms: Semi-automated tetranucleotide-frequency based emergent self-organizing maps (ESOMs, specifications in Probst et al., 2016), differential coverage ESOMs[9] (specifications in Probst et al., 2016), ABAWACA 1.00[10], ABAWACA 1.07 (https://github.com/CK7/abawaca), CONCOCT[11], Metabat[12] and Maxbin2[13] (automated binners were used with default settings). Best genomes from each sample were selected using DAS Tool[14] (https://github.com/cmks/DAS_Tool), which utilizes a scoring metric based on the number of bacterial and archaeal single copy genes and the existence of multiple single copy genes in a bin. Genomes with completeness >70% and less than 3 multiple single copy genes were selected from each sample and de-replicated at 98% nucleotide identity according to the scheme presented in Supplementary Figure 2. Representative genomes were curated using GC, coverage and taxonomy of each scaffold in the ggKbase environment[15] (http://ggkbase.berkeley.edu). For taxonomy information, we used the taxonomic information of each predicted protein of an in-house database and determined the taxonomic winner of each scaffold taking into account eight different levels of taxonomy (the lowest taxonomic level that has >50% of the proteins assigned to represents the taxonomy winner of a scaffold).

Estimation of genome completeness and contamination level. Single-copy genes of bacteria (51 in total) and archaea (38 in total) were used to estimate genome completeness (https://github.com/AJProbst/sngl_cp_gn)[8]. Contamination level was estimated by the presence of multiple copies of a single copy gene, excluding fragmentation of genes. The genes and the completeness

of the newly generated genomes from this study can be found in Supplementary Table 2. Only genomes with a completeness of at least 70% (medium-quality) were considered for further analysis.

Single cell genomics. On 10 April 2014, 1 liter of water from the minor eruption phase of the geyser cycle was collected in parallel to samples for metagenomics (Supplementary Figure 2). Sequential vacuum filtration onto a 3.0-µm and then a 0.2-µm polycarbonate membrane filter (EMD Millipore) was performed in order to fractionate cells. Folded filters were placed in cryotubes with a solution of 1-fold TE and 5.5% glycerol, frozen on dry ice, and stored at -80° C. On 21 August 2014, the same procedure was performed with water from the recovery phase of the geyser cycle (Supplementary Figure 2). Two liters of water were filtered through a 3.0-µm filter and 1 liter of the filtrate sequentially through a 0.8-µm and a 0.2-µm pore size membrane. Just prior to fluorescence-activated cell sorting, tubes were thawed at 4°C, gently vortexed, and filters were removed. Single cells were isolated from the resulting solution with FACS, lysed, and subjected to whole genome amplification (WGA) as previously described[16], with the following modifications: the alkaline lysis was preceded by a 20 min digest with $5x10^{-2}$ U of lysozyme (Epicentre) at 30° C; WGA was performed with a REPLI-g Single Cell Kit (Qiagen) with a scaled-down reaction volume of 2 nl; and the amplification reaction was incubated for 6 h at 30 °C. WGA reactions were diluted 10-fold, then aliquots were taken and further diluted 200-fold before PCR screening targeting the SSU rRNA (forward primer: 926wF (GAAACTYAAAKGAATTGRCGG) and reverse primer: 1392R (ACGGGCGGTGTGTRC)) using a QuantiNova SYBR Green PCR kit (Qiagen) for 45 cycles of amplification[17]. All SAGs with a positive PCR reaction were selected for shotgun sequencing. SAGs were sequenced at the DOE Joint Genome Institute (JGI) using Nextera libraries (Illumina) and the Illumina NextSeq platform following standard protocols (http://www.jgi.doe.gov). Quality filtering, trimming, error correction, and assembly of genomes was performed with the standard pipeline for single cells, which utilizes SPAdes version 3.8.2[18] for the uneven coverage of single-cell genomes, with parameters "--phred-offset 33 -t 16 -m 120 --sc --careful -k 25,55,95 –12". Genomes were assessed based on JGI standard quality control metrics for single cells (except the total assembly size cutoff), and subjected to additional rigorous decontamination. Genomes had their contigs individually binned by MetaBAT[12], and only primary bins at least 100 KB in size were retained and manually curated in ggKbase using GC and taxonomy information.

Quantitative digital droplet PCR (ddPCR). Species specific ddPCR was used to confirm relative abundance measures from metagenome sequencing for three organisms. Absolute abundances of 16S rRNA genes of the highly abundant genomes of *Ca.* "Altiarchaeum" (CG_4_9_14_0_8_um_filter_Altiarchaeum_SM1_32_20), *Sulfurimonas* sp. (CG07_land_8_20_14_0_80_Sulfurimonas_36_56), *Hydrogenophilaceae* (CG12_big_fil_rev_8_21_14_0_65_Hydrogenophilales_61_21) in Crystal Geyser samples were elucidated. The 16S rRNA gene region of each species was targeted with the following primers: *Ca.* "Altiarchaeum" with SM1_648f (5'-GACCATCTGGGCGAAGGC-3')[19] and SM1_825r (5'-CCCCAGACGGTGGACTTAAC-3'), *Sulfurimonas* sp. with Sulf_85f (5'-TATGATTAGTGGCGCACG-3') and Sulf_226r (5'-GGCCGATCTCTTAGCGAAA-3') and *Hydrogenophilaceae* with Hydro_f (5'-GGGTTGTAAACCGCTTTCGG-3') and Hydro_r (5'-CGATTAACGCTCGCACCCTA-3'), repsectively. New primers were designed off 16S rRNA genes assembled from metagenomic reads using NCBI's primer designing tool (https://www.ncbi.nlm.nih.gov/tools/primer-blast) and evaluated regarding specificity against all assembled metagenomes using blastn (-task blastn-short)[20]. These primers were used to set up positive controls of 16S rRNA genes for ddPCR. In brief, the 16S rRNA gene fragment was amplified from a CG

sample collected in 2015, cloned into *E. coli* (TOPO TA Cloning kit, Invitrogen, Carlsbad, US) and the insert of the purified vector was sequenced using Sanger sequencing technology (QB3-Berkeley, US). The identity of the sequenced 16S rRNA gene fragment was confirm using MUSCLE alignment[21] against the 16S rRNA gene of the genomic template.

Genomic DNA from each sample collected in 2015 (which was used for tracking organisms across the geyser cycle) was quantified using the Qubit high-sensitivity DNA assay (Invitrogen, Carlsbad, CA). 0.01X and 0.001X dilutions were made for samples below below the 10 ng/µl in concentration and for samples above or equal to 10ng/µl, respectively. PCR reactions were performed on the Bio-Rad QX200 platform using 12 µl of each dilution as the template, coupled with 0.25 µl of 10 µM forward and reverse primers (see above), and 12.5 µl of 2X ddPCR EvaGreen Supermix (Bio-Rad, Hercules, CA). Droplet generation was done using 20 µl of each mixture following the QX200 Droplet Generator protocol (Bio-Rad). Thermocycling parameters were set for primer pairs as follows: *Ca.* "Altiarchaeum": (1) 95˚C for 3 min, (2) 95˚C for 30 s, (3) 55˚C for 30 s, (4) 72˚C for 45 min, (5) 35 cycles (go to steps 2–4 x29), (6) 72˚C for 5 min, and (7) hold at 4˚C, *Sulfurimonas* sp.: (1) 95˚C for 3 min, (2) 95˚C for 30 s, (3) 48.5˚C for 60 s, (4) 72˚C for 1 min, (5) 30 cycles (go to steps 2–4 x29), (6) 72˚C for 10 min, and (7) hold at 4˚C, *Hydrogenophilaceae*: (1) 95˚C for 3 min, (2) 95˚C for 30 s, (3) 53.8˚C for 60 s, (4) 72˚C for 1 min, (5) 30 cycles (go to steps 2–4 x29), (6) 72˚C for 10 min, and (7) hold at 4˚C. DPEC-treated water was used as template for negative controls, vectors of plasmids (see above) were used as positive controls. Reactions were performed in duplicates or triplicates for all samples and species (technical replicates); sample position was randomized for each droplet reading. Quantitative ddPCR data was analyzed using the QuantaSoft software package (Bio-Rad). The thresholds separating negative and positive partitions were set just before the negative population. Values of technical replicates were averaged to retrieve absolute abundances of 16S rRNA genes for each of the three species. These were correlated with the relative abundances generated using metagenome sequence mapping (Pearson correlation)[22].

Scanning electron microscopy. After filtration of the Crystal Geyser groundwater onto 0.22-µm filters (Isopore disc PC philic, Millipore), cells were directly fixed on the filters using 2.5% glutaraldehyde in 75 mM cacodylate buffer also containing 2 mM $MgCl_2$. Following post-fixation with 1% OsO_4 for 100 min, these samples were dehydrated in a graded acetone series and finally critical-point-dried. To enhance conductivity, the fixed and dehydrated filters were mounted onto aluminum stubs and sputter-coated for 40s with platinum. Subsequent scanning electron microscopy was carried out with a Hitachi S-4100 SEM (Hitachi, Tokyo, Japan). In several SEM micrographs, vertical lines are visible within the image. As we used the images as they are without further processing, these lines are not the result of image stitching. They are caused by a device called DigiScan, which is responsible for the generation of black/white images from SE (secondary electron) signals from the detector. Due to the high age of 25 years of the DigiScan, it is producing these vertical line imaging artifacts at different positions when the ratio of scan speed and image dwell time is not ideal.

2. Supplementary Figures

Supplementary Fig. 1 | Details of the geochemical and hydrogeological interpretation of Crystal Geyser water.

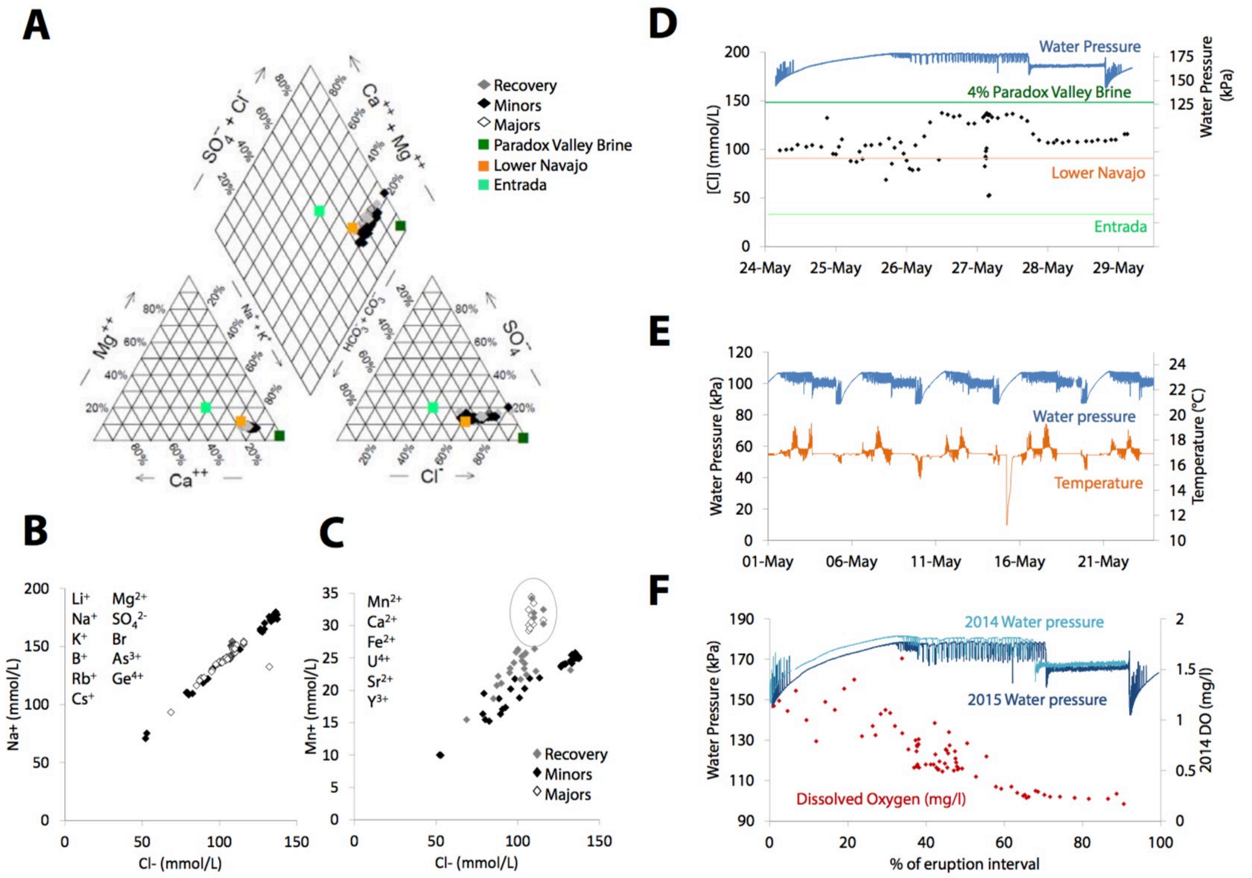

A. Piper plot of Crystal Geyser water geochemical compositions in relation to groundwater sourced from Entrada Sandstone, Lower Navajo Sandstone, Paradox Valley Brine[23], and Green River compositions (sampled in this study). The proximity of Crystal Geyser water samples to Lower Navajo groundwater suggests that water from this stratigraphic region is a dominant water source in Crystal Geyser discharge.
B - C. Sample plots of major ion and metal concentrations vs. chloride concentration for Crystal Geyser discharge, coded according to geyser phases. **B.** Most of the ions (not plotted but listed in top right corner of the plot) analysed in Crystal Geyser water plotted against chloride in a similar fashion as Na vs. Cl (plotted, showing a linear mixing line between Paradox Valley Brine composition[24] (Cl^- ~ 3600 mmol/l; not shown) and meteoric water (graph origin), consistent with mixing of meteoric water with brine). The highest chloride concentrations occurred in the Minors, suggesting a slightly greater contribution of Paradox Valley Brine during this phase. **C.** The concentrations of six of the elements measured (Mn, Ca, Fe, U, Sr, and Y) were distinctly elevated during the major eruption phase. These elements occur in dolomite, calcite, and hematite, all minerals found in the Entrada Sandstone[23] suggesting a larger contribution from the Entrada Sandstone during the major phase as also reported elsewhere[25].
D. Time series chloride concentrations in Crystal Geyser water samples plotted in relation to chloride concentrations measured in Entrada and Navajo Sandstone goundwaters (shown as lines)[23]. Green horizontal line showing chloride concentration equivalent to ~4% Paradox Valley brine represents some

deeper formation water[24]. Crystal Geyser chloride concentrations increased during the minor eruption phase, suggesting increased contribution from deeper groundwater.

E. Monthly temperature recorded in May 2015, the month of the present study's main sampling campaign. Temperature varies around a baseline temperature of 16.9 degrees Celsius. Using the local geothermal gradient of 21.2°C/km and mean annual air temperature as the recharge temperature (7°C to 10°C)[26], water in Crystal Geyser rises from 320-460 meters. At Crystal Geyser, the upper limit of this depth corresponds with the base of the Navajo aquifer, while the lower limit corresponds with the Wingate aquifer[23]. Given the consistency with the geochemical data, we conclude that the Navajo aquifer is the main source of groundwater considering the entire eruption cycle.

F. Although O_2 concentrations were below detection limit on the gas chromatography, genomic data showed a clear influence of O_2 on the microbial community. Thus, archived data from a 2014 field campaign were re-investigated, which included dissolved oxygen (DO) measurements collected using a YSI multi-parameter water quality sonde submerged in a bucket overflowing with geyser discharge water during water sampling. The highest dissolved oxygen concentrations (~1.5 mg/l) were observed at the end of the recovery period, after the water column slowing recovered ~3 m over 1.5 days with exposure to atmospheric oxygen at the top of the water column and in the shallow groundwater recirculation (i.e. recently discharged surface water in the geyser pool travelling back into the borehole via shallow cracks that are visible in the near-surface borehole casing). The dissolved oxygen concentrations decreased throughout the minor phase and were lowest in the major phase (~0.25 mg/l), when Crystal Geyser's discharge rate was great enough to prevent shallow recirculation of erupted water.

Interpretation: Geochemical (panel A-C) and baseline temperature 16.9°C (panel D) data show that overall, Crystal Geyser's discharge water composition is primarily sourced from the Navajo Sandstone. An increased contribution comes from the shallower Entrada Sandstone aquifer during the major eruption phase (B) and an increased contribution from deeper formations (possibly the lower Wingate or White Rim Sandstone, and interpreted as increased Paradox brine by others[25]) during the minor eruption phase (C) were observed. Panel E shows varying O_2 levels with phase of eruption, which influences the microbial communities sampled from Crystal Geyser.

Replication: We performed 7250 measurements for EC, temperature, and water pressure; of these, 2330 were performed during the recovery, 2820 were performed during in minor eruptions, 1560 were performed during the major eruption, and 540 were performed during the following recovery. No technical replicates were performed. We analyzed 76 water samples for major ions and trace metals; of these, 28 were sampled during recovery, 37 were sampled during minor eruptions, and 11 were sampled during the major eruption. Technical replicates: Of the 28 samples from recovery, 4 samples were replicated once. Of the 37 samples from minor eruptions, 5 samples were replicated once. Of the 11 samples from the major eruption, three samples were replicated once.

Additionally, data from a 2014 field campaign is included in panel F. In 2014, we took 4512 measurements of water pressure; of these, 1887 were performed during the recovery, 1476 were performed during minor eruptions, and 1149 were performed during the major eruption. No technical replicates were performed. We also measured dissolved oxygen in 78 water samples; of these, 32 were performed during the recovery, 37 were performed during in minor eruptions, and 9 were performed during the major eruption. No technical replicates were performed.

Supplementary Fig. 2 | Study design and workflow.

We generated 30574 genomes from metagenomes using the seven binning algorithms. From these, DAS Tool identified 5795 unique bins, 2216 were of high quality (>70% completeness). These genomes were dereplicated by sampling day and the best representative (most complete, lowest contamination) was used for manual curation in ggKbase (based on GC, taxonomy and coverage of the respective sample). The remaining 986 genomes were curated using ra2, which removed two genomes due to low coverage information. The final 984 genomes were combined with 222 genomes from a previous study and checked against the generated single cell genomes, which enabled the recruitment of another two genomes, resulting in 1217 genomes in total. *manual binning method

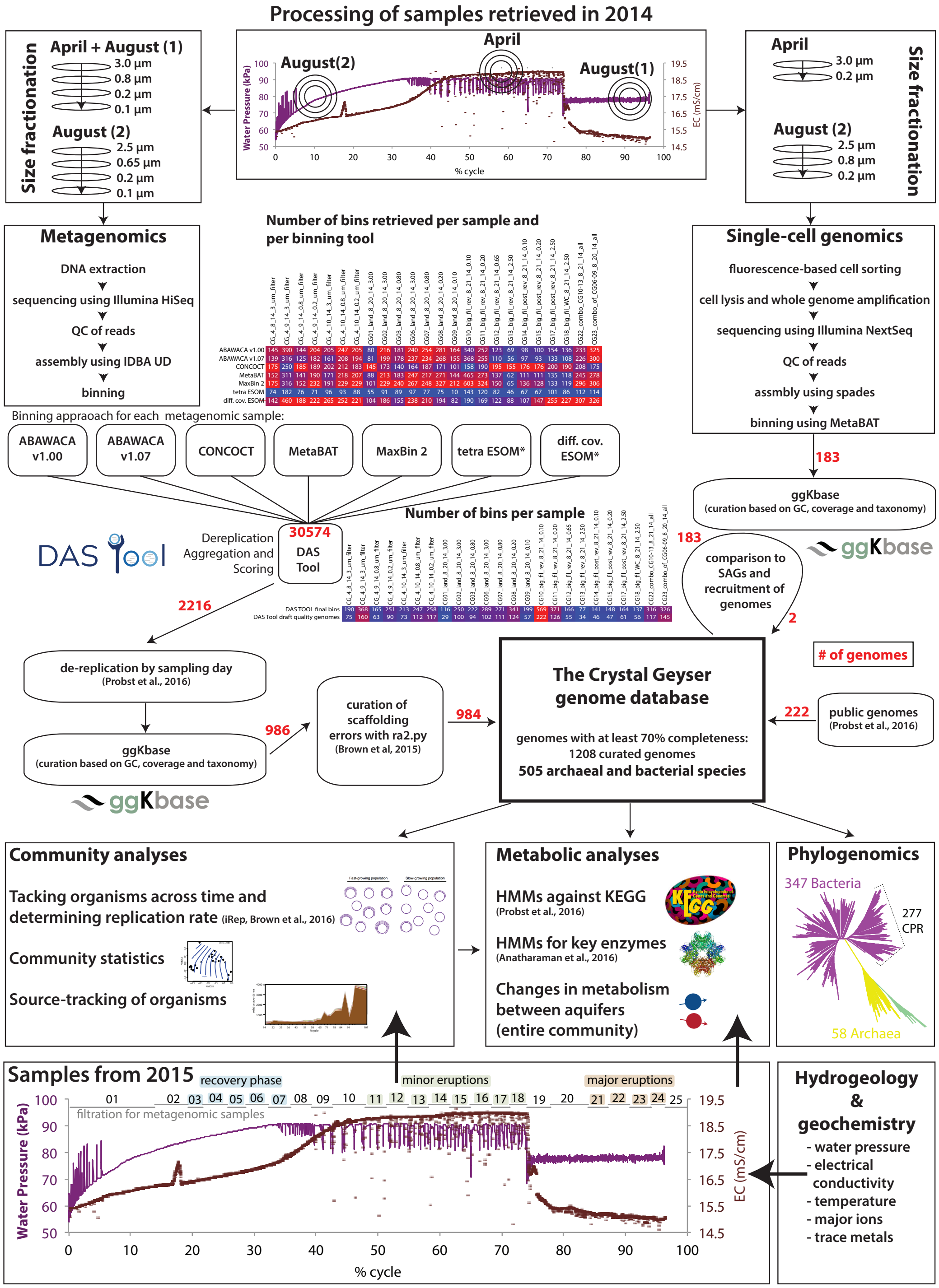

Supplementary Fig. 3 | Comparison of SAGs to genomes from metagenomes. Two examples, a 100% complete genome from metagenome against a SAG (B), and the best SAG (90% complete) against its corresponding genome from metagenome (C) are shown in detail regarding their alignment rate. The two draft quality SAGs (>70% completeness) that did not have a corresponding genome from metagenome were compared to the corresponding metagenomes of the collected filters. CG_SAG_2014_w16S_Bacteria_49_1 was neither detected in the assembled metagenome based on rpS3 sequences nor detectable via read mapping (bowtie2, --sensitive) in the corresponding metagenome (CG13_big_fil_rev_8_21_14_2.50). CG_SAG_2014_w16S_Bacteria2_36_1 was detected in the assembled metagenome based on rpS3 protein similarity (100% identity). The dataset is based on 183 high quality SAGs compared to 983 genomes from metagenomes.

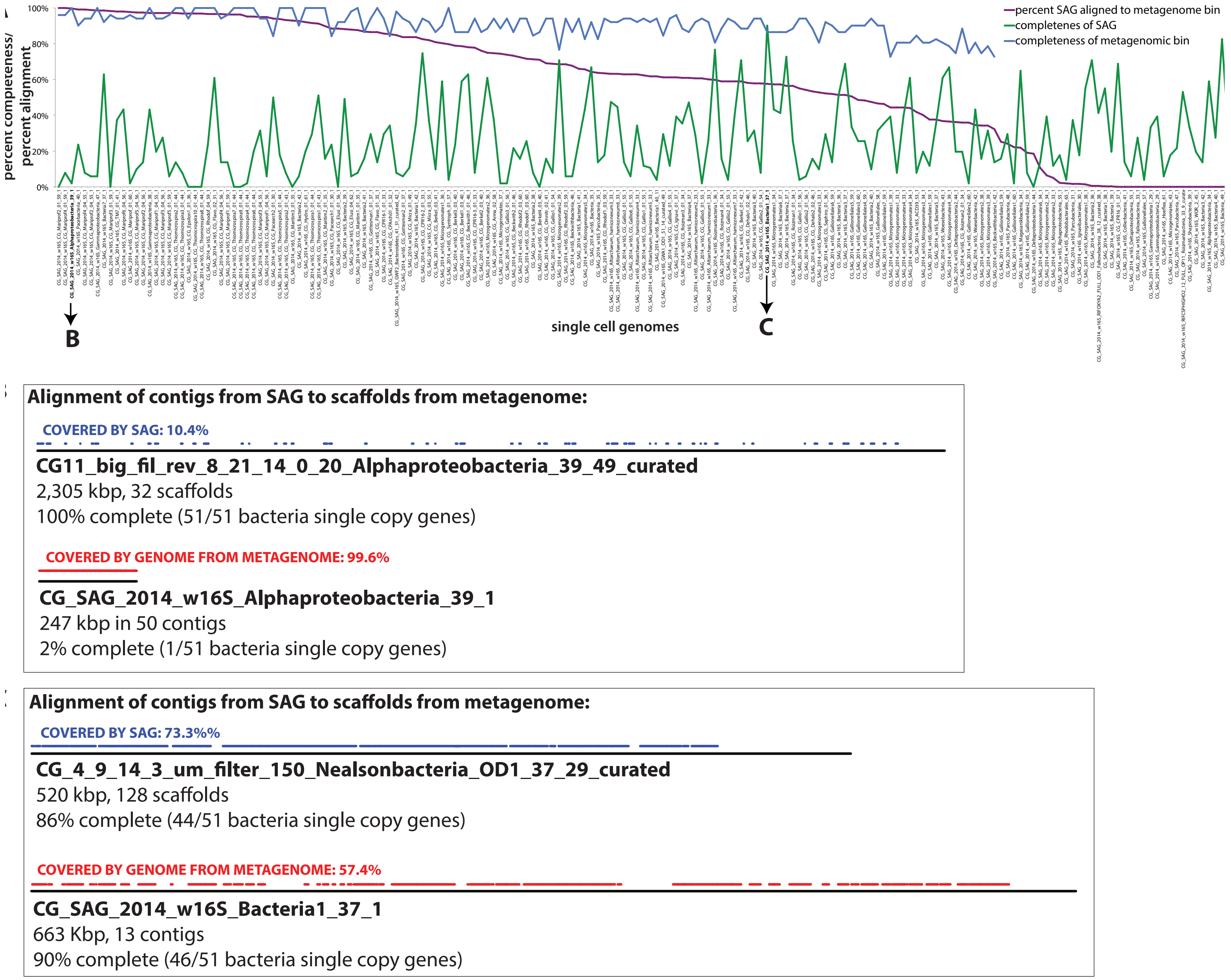

Supplementary Fig. 4 | Rank abundance curve to demonstrate the theoretically possible community coverage with metagenomic sequencing for a single sample (CG04) in 2015 (using 1224 different rpS3 genes from 24 samples taken in 2014).

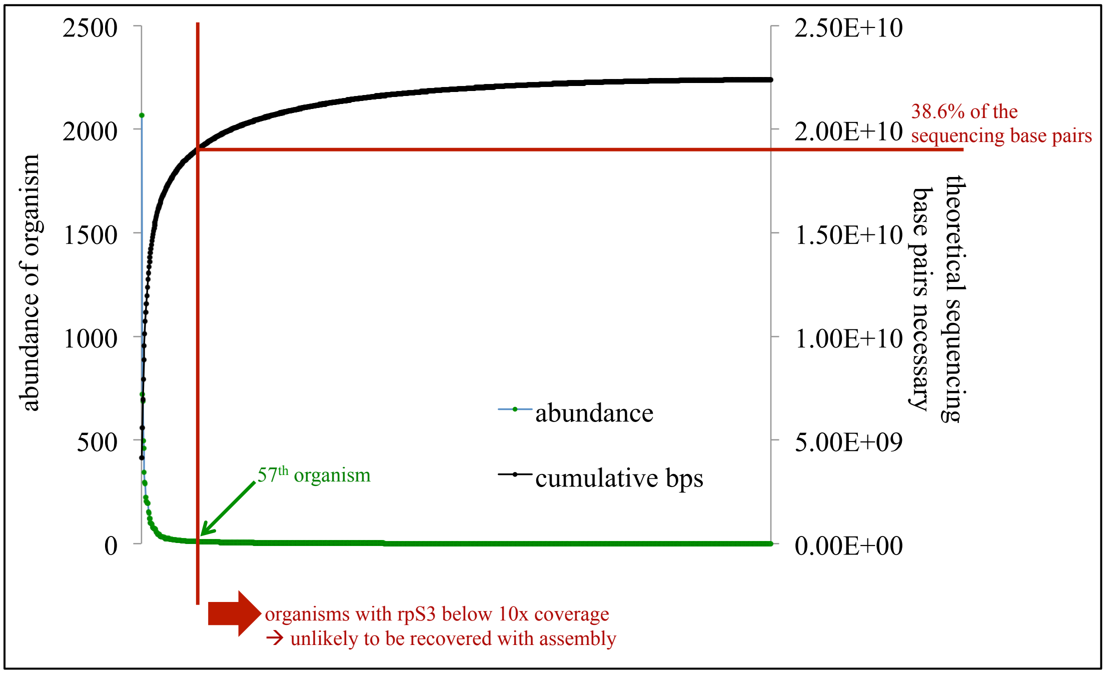

In order to determine the theoretical coverage of genomes that can be generated from a metagenomic sequencing, we extracted all rpS3 genes (using HMMs built from a previous dataset[27]), clustered them at 98% amino acid sequence similarity[28] (representative at species level) and stringently mapped reads (bowtie2 "--sensitive")[29] of the 2015 sample CG04 to the longest scaffolds of each rpS3 cluster to determine the coverage.

Coverage of each rpS3 gene was plotted as a rank abundance curve (green) and the theoretical sequencing base pairs (black) were calculated assuming an average genome size of 2 Mbps (coverage X genome size). Since genomes with less than 10X coverage cannot be properly assembled from metagenomic data, about 57 organisms from that particular sample would be recoverable. This accounts for 38.6% of the sequencing reads and provides evidence that the majority of reads go into sequencing of low abundant organisms. Although this theoretical assessment suffers from a standardized genome size and assembled rpS3 sequences, it demonstrates that the average mapping rate of ~50% against the 505 genomes (Supplementary Table 4) accounts for the majority of organisms present in the Crystal Geyser community.

Supplementary Figure 5. Scanning electron microscopy images of diverse microorganisms retrieved from Crystal Geyser groundwater. Five samples were taken at three different time points of the geyser (n=5) and the different morphologies observed are displayed in these images. **A**. Filamentous microorganisms, **B**.-**F**. rod-shaped microorganisms, **G**. spherical cell which can clearly differentiated from *Ca.* "Altiarchaeum" due to missing cell surface appendages and a smooth cell surface (compare Supplementary Figure 9), **H**. microscopic structures as they have been described for iron-oxidizing bacteria like Zetaproteobacteria[30], which have been identified in Crystal Geyser (Figure 2). For morphology of *Ca.* "Altiarchaeum", the most dominant organism in the geyser fluids, please see Supplementary Figure 9.

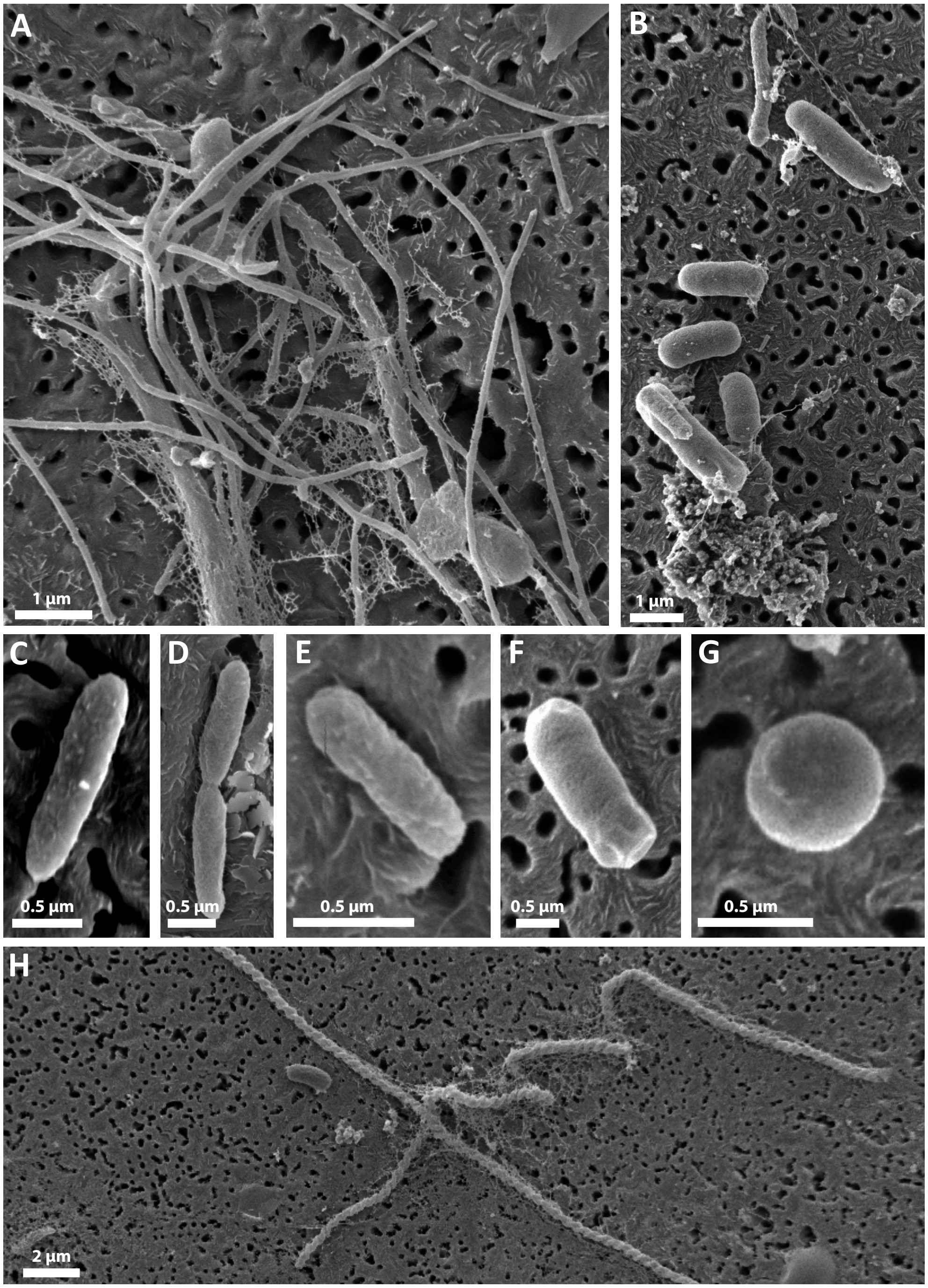

Supplementary Figure 6. Comparison of relative abundance measure from genome resolved-metagenomics mapping with quantitative digital droplet PCR (ddPCR). While the mapping approach tracked entire genomes, the ddPCR was designed for tracking 16S rRNA genes. Thus, the absolute 16S rRNA gene abundance is not comparable between species (e.g. the tracked *Sulfurimonas* seemed to have multiple copies of the same 16S rRNA gene). Agreement of the two methods was determined via linear correlation of the relative abundance measures and the absolute 16S rRNA gene abundance for each species individually (Pearson correlation, number of biological replicates was 24). Technical replicates of each ddPCR reaction are given as italic numbers at the bottom of the figure panels. Metagenomics abundance estimates were not replicated. Colors of the different phases of the geyser are analogous to main Figure 3.

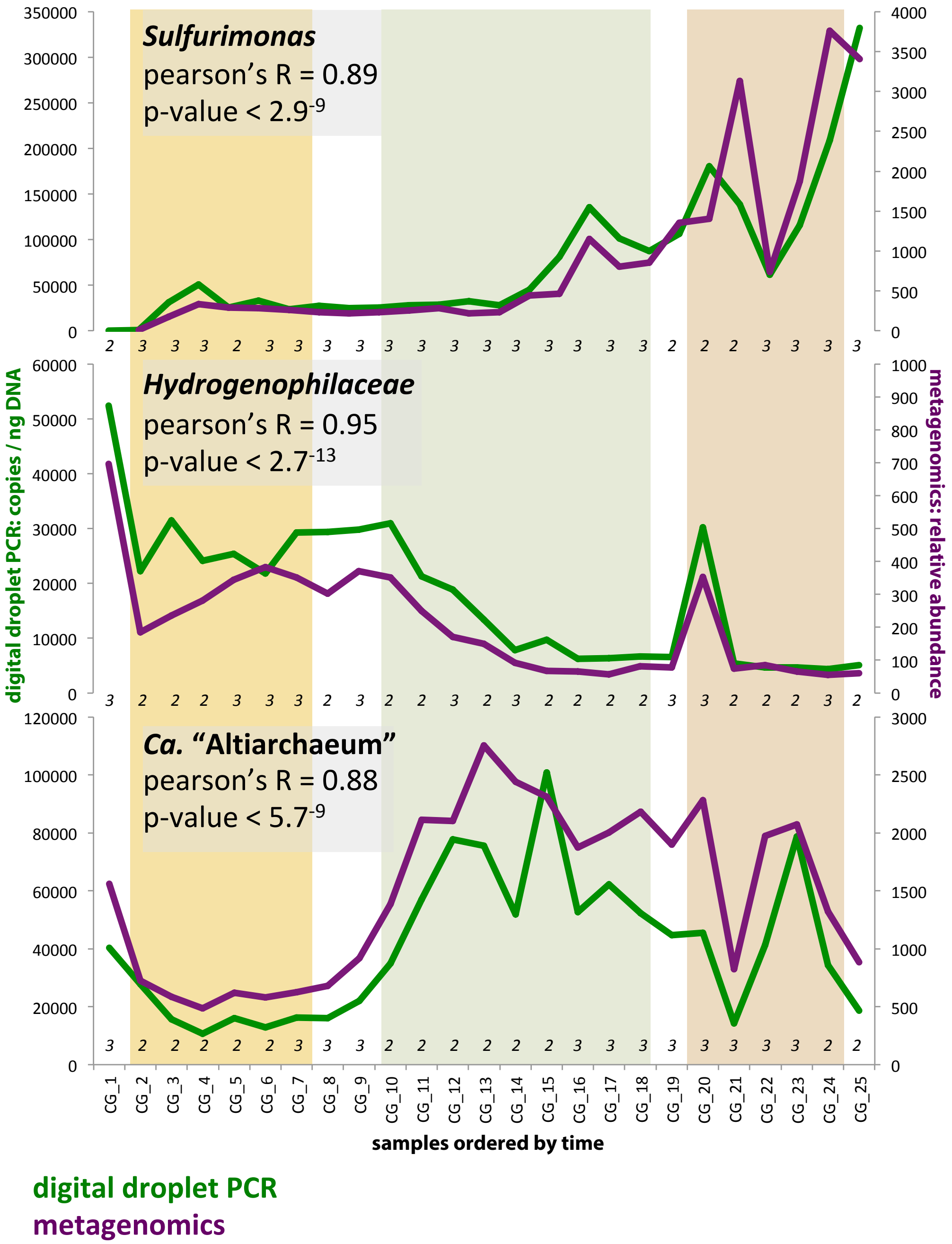

Supplementary Fig. 7 | Boxplot and ANOVA of total abundance of Archaea across the three aquifers. Table in the upper corner displays p-values of ANOVA followed by a Tukey HSD between the three groups (aquifers) tested. Numbers of samples in recovery phase, minor eruptions and major eruptions were five, eight, and four (biological replicates).

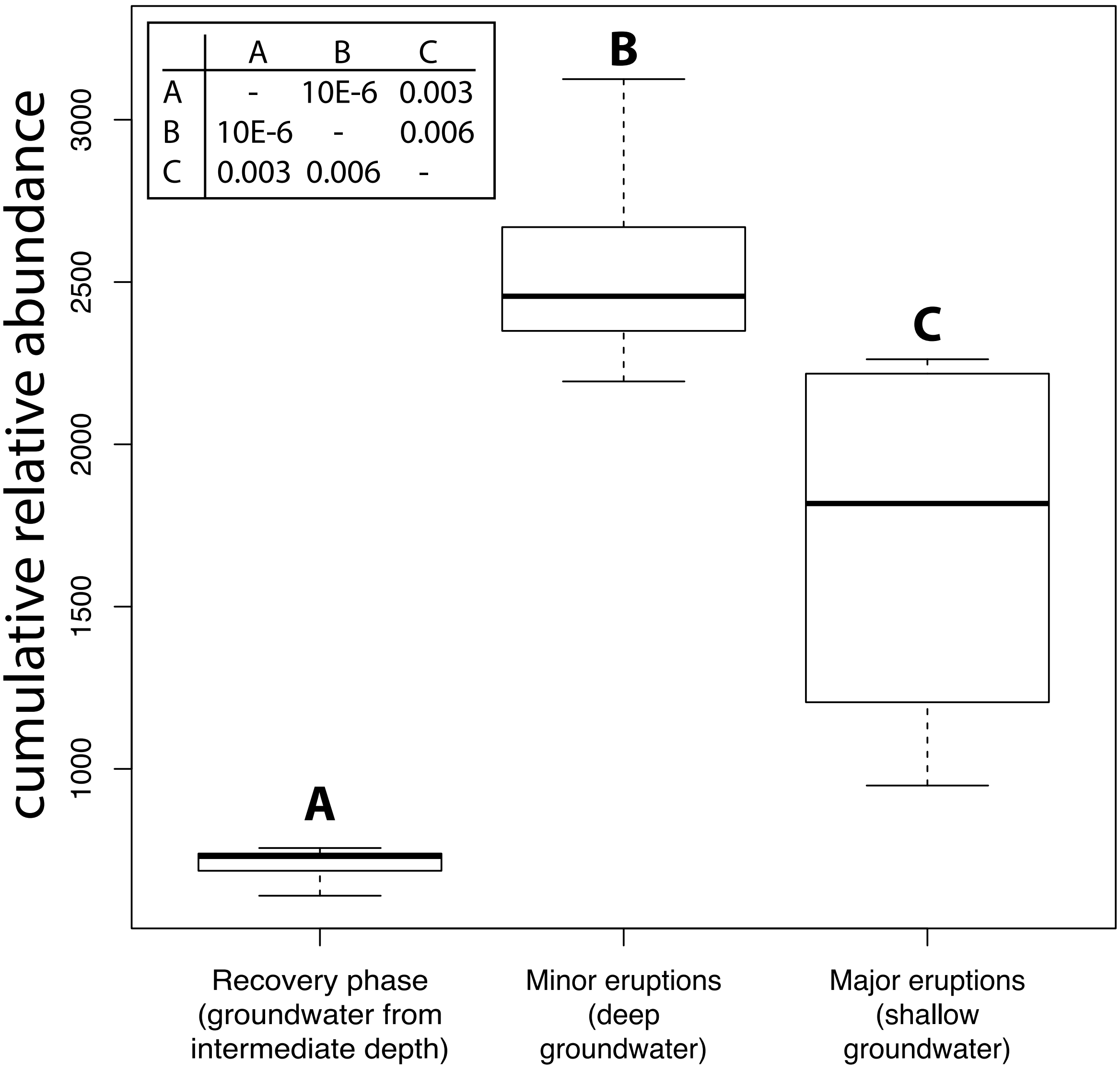

Supplementary Fig. 8 | Cys-rich surface protein encoded in *Ca.* "Huberarchaeum crystalense". The predicted function of the protein based on interpro is the binding of calcium ions. This function is also present in hemolysins, which have the function to rupture cell membranes. This protein might be involved in opening up the host membrane to get access to its metabolites.

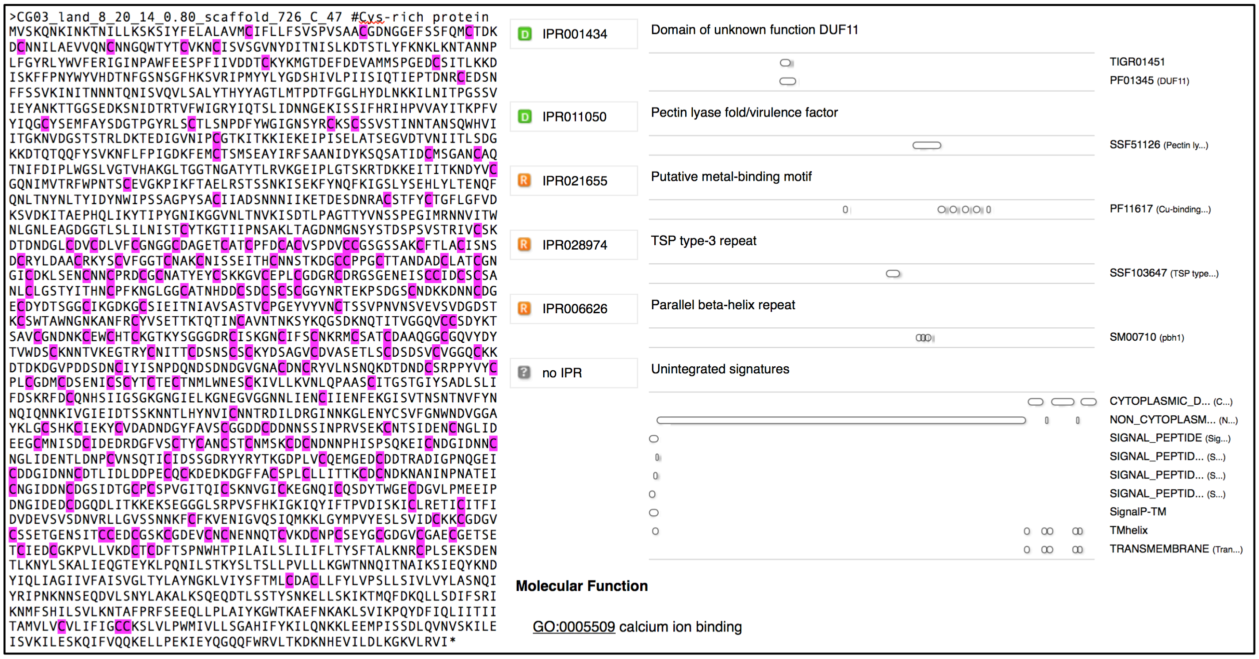

Supplementary Figure 9. Scanning electron micrographs of *Ca.* "Altiarchaeum" cells. Identification was possible due to the specific cell size and cell architecture, which included a rough cell surface and many cell surface appendages (compare to previous publications[31,32]). Five samples were taken at three different time points of the geyser (n=5) and *Ca.* "Altiarchaeum" was most frequently observed in samples retrieved during the minor eruptions, which is in accordance with metagenomic and quantitative ddPCR results. **A**. Cell of *Ca.* "Altiarchaeum" with spherical attachment, which could potentially be its putative symbiont *Ca.* "Huberarchaeum crystalense" (also see Figure 6). **B**.-**C**. Cells of *Ca.* "Altiarchaeum", whereas the cell in C is undergoing cell division, which is indicative of cellular activity of *Ca.* "Altiarchaeum" at the moment of sampling. **D**. Multiple connected cells of *Ca.* "Altiarchaeum" demonstrating their high cellular abundance in groundwater samples collected at Crystal Geyser.

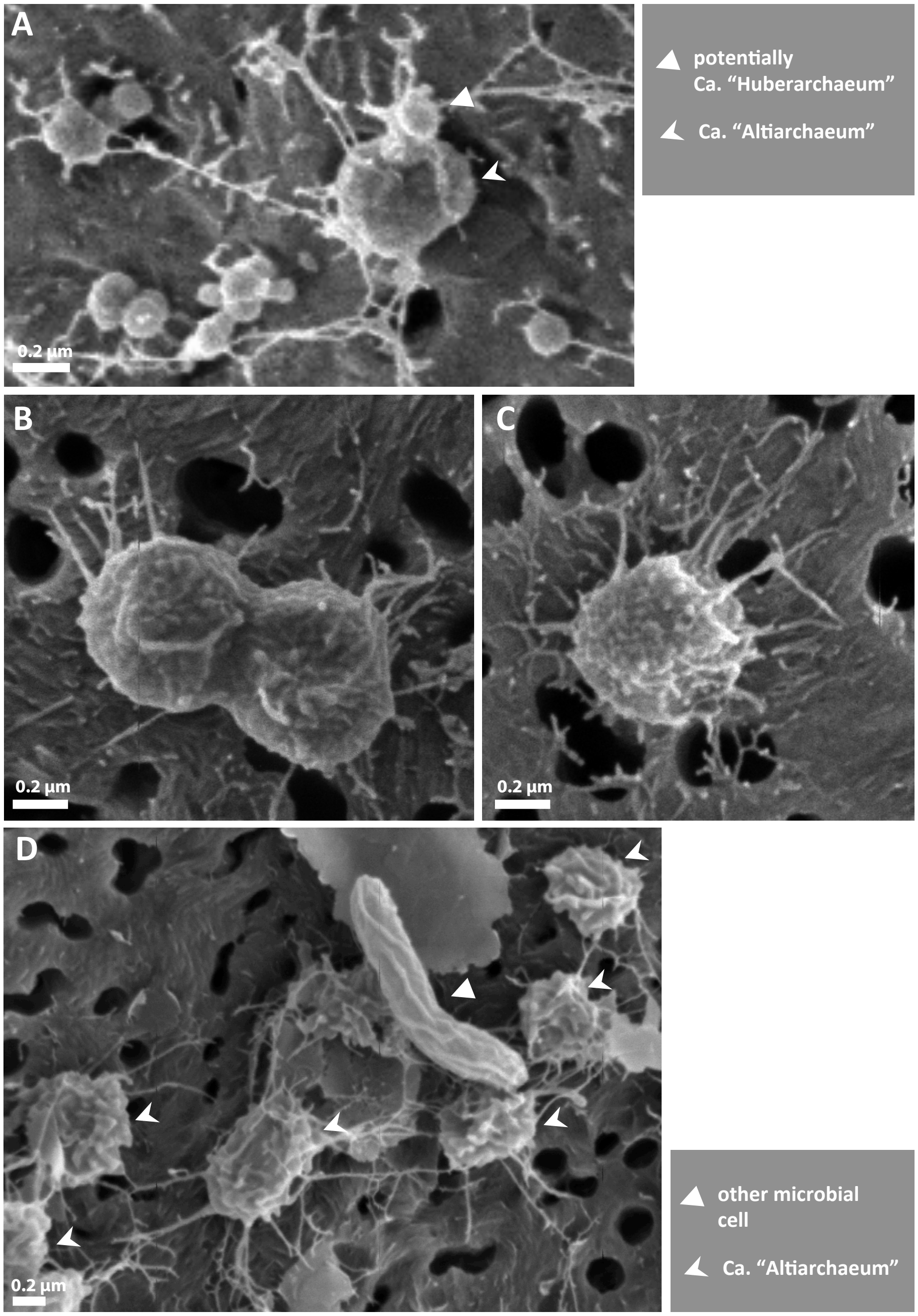

3. Supplementary Tables

Supplementary Table 1 | Sample overview. All samples had a read length or were trimmed to a read length of 150 bps.

Supplementary Table 2 | Genome completeness of 983 genomes from metagenomes and 183 single cell genomes based on 51 bacterial single copy genes and 38 archaeal single copy genes.

Supplementary Table 3 | Overview of the taxonomy of the 505 genomes and novel phylum names that have a *Candidatus* status.

Supplementary Table 4 | Community coverage based on read-mapping of 2015 samples. Calculations are based on sensitive bowtie2 mapping ("--sensitive")[29]. Median percent coverage was 48.11%.

sample	% read coverage	sample	% read coverage	sample	% read coverage
CG01	45.31%	CG10	49.86%	CG18	49.53%
CG02	53.71%	CG11	51.50%	CG19	45.53%
CG03	47.41%	CG12	53.56%	CG20	42.82%
CG04	48.12%	CG13	53.09%	CG21	31.04%
CG05	46.62%	CG14	54.48%	CG22	45.22%
CG06	45.94%	CG15	54.86%	CG23	42.74%
CG07	46.96%	CG16	48.65%	CG24	34.38%
CG08	48.11%	CG17	51.93%	CG25	36.18%
CG09	48.60%				

Supplementary Table 5 | Normalized relative abundance of organisms from Crystal Geyser across the 25 metagenome samples (see Supplementary Table 3). Relative abundance values are based on bowtie2[29] mapping allowing a maximum of three mismatches per read (98% identity; see methods). Column B, C, and D indicate where the respective organism was enriched.

Supplementary Table 6 | Overview of the environmental variables collected for each metagenomic sample. Continuously collected data for variables like temperature were averaged over the sampling time, in which the metagenomic sample was acquired. BioENV was performed on all continuous variables listed, except sampling date.

Supplementary Table 7 | Metabolic profile of 505 species detected at Crystal Geyser based on recovered genomic content.

Supplementary Table 8 | iRep[33] values of organisms across the different metagenomes of the geyser cycle. iRep analysis is based on mapping reads using bowtie2[29] allowing one mismatch per read.

4. Supplementary Files

Supplementary File 1 | Phylogenetic tree based on 16 concatenated ribosomal proteins (details see methods).

Supplementary File 2 | HMM profile of all 11 genomes of *Candidatus* "Huberarchaeum crystalense". The completeness of each pathway is displayed and the individual enzymes for each KEGG module are displayed by clicking onto the respective numbers (counts) of each module. The displayed predictions were retrieved via HMM search against each single KEGG enzyme with e-values < E-10 as described in the methods. For details on HMM generation please see Probst et al., 2016.

5. References

1. Anonymous. *Progress made at Utah Southern and Ruby Oil well tests*. Times Independent Newspaper. (1936).
2. Barton, J. R. & Fuhriman, D. K. *Crystal Geyser Project: A Study of Some Alternative Methods for Eliminating the Salt Contribution of Crystal Geyser from the Green River*. (Center for Environmental Studies, Brigham Young University, 1973).
3. Baer, J. L. & Rigby, J. K. Geology of the Crystal Geyser and environmental implications of its effluent, Grand County, Utah. *Utah Geol.* **5,** 125–130 (1978).
4. Emerson, J. B., Thomas, B. C., Alvarez, W. & Banfield, J. F. Metagenomic analysis of a high carbon dioxide subsurface microbial community populated by chemolithoautotrophs and bacteria and archaea from candidate phyla. *Environ. Microbiol.* **18,** 1686–1703 (2016).
5. Peng, Y., Leung, H. C., Yiu, S.-M. & Chin, F. Y. IDBA-UD: a de novo assembler for single-cell and metagenomic sequencing data with highly uneven depth. *Bioinformatics* **28,** 1420–1428 (2012).
6. Cui, X., Lu, Z., Wang, S., Jing-Yan Wang, J. & Gao, X. CMsearch: simultaneous exploration of protein sequence space and structure space improves not only protein homology detection but also protein structure prediction. *Bioinformatics* **32,** i332–i340 (2016).
7. Lowe, T. M. & Eddy, S. R. tRNAscan-SE: a program for improved detection of transfer RNA genes in genomic sequence. *Nucleic Acids Res.* **25,** 955–964 (1997).
8. Probst, A. J. *et al.* Genomic resolution of a cold subsurface aquifer community provides metabolic insights for novel microbes adapted to high CO_2 concentrations. *Environ. Microbiol.* **19**(2), 459-474 (2017).
9. Sharon, I. *et al.* Time series community genomics analysis reveals rapid shifts in bacterial species, strains, and phage during infant gut colonization. *Genome Res.* **23,** 111–120 (2013).
10. Brown, C. T. *et al.* Unusual biology across a group comprising more than 15% of domain Bacteria. *Nature* **523,** 208–211 (2015).
11. Alneberg, J. *et al.* Binning metagenomic contigs by coverage and composition. *Nat. Methods* **11,** 1144–1146 (2014).
12. Kang, D. D., Froula, J., Egan, R. & Wang, Z. MetaBAT, an efficient tool for accurately reconstructing single genomes from complex microbial communities. *PeerJ* **3,** e1165 (2015).
13. Wu, Y.-W., Tang, Y.-H., Tringe, S. G., Simmons, B. A. & Singer, S. W. MaxBin: an automated binning method to recover individual genomes from metagenomes using an expectation-maximization algorithm. *Microbiome* **2,** 26 (2014).
14. Sieber, C. M. *et al.* Recovery of genomes from metagenomes via a dereplication, aggregation, and scoring strategy. *bioRxiv* 107789 (2017).
15. Wrighton, K. C. *et al.* Fermentation, Hydrogen, and Sulfur Metabolism in Multiple Uncultivated Bacterial Phyla. *Science* **337,** 1661–1665 (2012).

16. Rinke, C. *et al.* Obtaining genomes from uncultivated environmental microorganisms using FACS–based single-cell genomics. *Nat. Protoc.* **9,** 1038–1048 (2014).
17. Rinke, C. *et al.* Insights into the phylogeny and coding potential of microbial dark matter. *Nature* **499,** 431–437 (2013).
18. Bankevich, A. *et al.* SPAdes: a new genome assembly algorithm and its applications to single-cell sequencing. *J. Comput. Biol.* **19,** 455–477 (2012).
19. Rudolph, C., Wanner, G. & Huber, R. Natural communities of novel archaea and bacteria growing in cold sulfurous springs with a string-of-pearls-like morphology. *Appl. Environ. Microbiol.* **67,** 2336–2344 (2001).
20. Altschul, S. F., Gish, W., Miller, W., Myers, E. W. & Lipman, D. J. Basic local alignment search tool. *J. Mol. Biol.* **215,** 403–410 (1990).
21. Edgar, R. C. MUSCLE: multiple sequence alignment with high accuracy and high throughput. *Nucleic Acids Res.* **32,** 1792–1797 (2004).
22. R Core, T. R: A language and environment for statistical computing. *Online http://www.r-proj.org* (2016).
23. Kampman, N. *et al.* Scientific drilling and downhole fluid sampling of a natural CO_2 reservoir, Green River, Utah. *Sci. Drill.* **16,** 33–43 (2013).
24. Rosenbauer, R. J., Bischoff, J. L. & Kharaka, Y. K. Geochemical effects of deep-well injection of the Paradox Valley brine into Paleozoic carbonate rocks, Colorado, USA. *Appl. Geochem.* **7,** 273–286 (1992).
25. Han, W. S. *et al.* Periodic changes in effluent chemistry at cold-water geyser: Crystal geyser in Utah. *J. Hydrol.* **550,** 54–64 (2017).
26. Heath, J. E., Lachmar, T. E., Evans, J. P., Kolesar, P. T. & Williams, A. P. Hydrogeochemical Characterization of Leaking, Carbon Dioxide-Charged Fault Zones in East-Central Utah, With Implications for Geologic Carbon Storage. *Carbon Sequestration Its Role Glob. Carbon Cycle* 147–158 (2009).
27. Hug, L. A. *et al.* A new view of the tree of life. *Nat. Microbiol.* **1,** 16048 (2016).
28. Edgar, R. C. Search and clustering orders of magnitude faster than BLAST. *Bioinformatics* **26,** 2460–2461 (2010).
29. Langmead, B. & Salzberg, S. L. Fast gapped-read alignment with Bowtie 2. *Nat. Methods* **9,** 357–359 (2012).
30. Chiu, B. K., Kato, S., McAllister, S. M., Field, E. K. & Chan, C. S. Novel Pelagic Iron-Oxidizing Zetaproteobacteria from the Chesapeake Bay Oxic–Anoxic Transition Zone. *Front. Microbiol.* **8,** (2017).
31. Probst, A. J. *et al.* Biology of a widespread uncultivated archaeon that contributes to carbon fixation in the subsurface. *Nat. Commun.* **5,** 5497 (2014).
32. Probst, A. J. *et al.* Coupling genetic and chemical microbiome profiling reveals heterogeneity of archaeome and bacteriome in subsurface biofilms that are dominated by the same archaeal species. *PloS One* **9,** e99801 (2014).
33. Brown, C. T., Olm, M. R., Thomas, B. C. & Banfield, J. F. Measurement of bacterial replication rates in microbial communities. *Nat. Biotechnol.* (2016).
