# Supplementary material for: Differential depth distribution of microbial function and putative symbionts through sediment-hosted aquifers in the deep terrestrial subsurface
Source: Nat Microbiol. 2018 Jan 29;3(3):328–36. doi: 10.1038/s41564-017-0098-y (PMC6792436; doi:10.1038/s41564-017-0098-y)
Supplement: Supplementary file 4 — HMM profile of all 11 genomes of Candidatus ’Huberarchaeum crystalense’. The completeness of each pathway is displayed and the individual enzymes for each KEGG module are displayed by clicking onto the respective numbers (counts) of each module. The displayed predictions were retrieved via HMM search against each single KEGG enzyme with e-values < E-10 as described in the methods. For details on HMM generation please see Probst et al., 2016. [file 41564_2017_98_MOESM4_ESM.html]

 
KEGG Pathway analysis output
 


|  |  |  |  |  |  |  |  |  |  |  |  |  |  |  |  |  |  |  |  |  |  |  |  |  |  |  |  |  |  |  |  |  |  |  |  |  |  |  |  |  |  |  |  |  |  |  |  |  |  |  |  |  |  |  |  |  |  |  |  |  |  |  |  |  |  |  |  |  |  |  |  |  |  |  |  |  |  |  |  |  |  |  |  |  |  |  |  |  |  |  |  |  |  |  |  |  |  |  |  |  |  |  |  |  |  |  |  |  |  |  |  |  |  |  |  |  |  |  |  |  |  |  |  |  |  |  |  |  |  |  |  |  |  |  |  |  |  |  |  |  |  |  |  |  |  |  |  |  |  |  |  |  |  |  |  |  |  |  |  |  |  |  |  |  |  |  |  |  |  |  |  |  |  |  |  |  |  |  |  |  |  |  |  |  |  |  |  |  |  |  |  |  |  |  |  |  |  |  |  |  |  |  |  |  |  |  |  |  |  |  |  |  |  |  |  |  |  |  |  |  |  |  |  |  |  |  |  |  |  |  |  |  |  |  |  |  |  |  |  |  |  |  |  |  |  |  |  |  |  |  |  |  |  |  |  |  |  |  |  |  |  |  |  |  |  |  |  |  |  |  |  |  |  |  |  |  |  |  |  |  |  |  |  |  |  |  |  |  |  |  |  |  |  |  |  |  |  |  |  |  |  |  |  |  |  |  |  |  |  |  |  |  |  |  |  |  |  |  |  |  |  |  |  |  |  |  |  |  |  |  |  |  |  |  |  |  |  |  |  |  |  |  |  |  |  |  |  |  |  |  |  |  |  |  |  |  |  |  |  |  |  |  |  |  |  |  |  |  |  |  |  |  |  |  |  |  |  |  |  |  |  |  |  |  |  |  |  |  |  |  |  |  |  |  |  |  |  |  |  |  |  |  |  |  |  |  |  |  |  |  |  |  |  |  |  |  |  |  |  |  |  |  |  |  |  |  |  |  |  |  |  |  |  |  |  |  |  |  |  |  |  |  |  |  |  |  |  |  |  |  |  |  |  |  |  |  |  |  |  |  |  |  |  |  |  |  |  |  |  |  |  |  |  |  |  |  |  |  |  |  |  |  |  |  |  |  |  |  |  |  |  |  |  |  |  |  |  |  |  |  |  |  |  |  |  |  |  |  |  |  |  |  |  |  |  |  |  |  |  |  |  |  |  |  |  |  |  |  |  |  |  |  |  |  |  |  |  |  |  |  |  |  |  |  |  |  |  |  |  |  |  |  |  |  |  |  |  |  |  |  |  |  |  |  |  |  |  |  |  |  |  |  |  |  |  |  |  |  |  |  |  |  |  |  |  |  |  |  |  |  |  |  |  |  |  |  |  |  |  |  |  |  |  |  |  |  |  |  |  |  |  |  |  |  |  |  |  |  |  |  |  |  |  |  |  |  |  |  |  |  |  |  |  |  |  |  |  |  |  |  |  |  |  |  |  |  |  |  |  |  |  |  |  |  |  |  |  |  |  |  |  |  |  |  |  |  |  |  |  |  |  |  |  |  |  |  |  |  |  |  |  |  |  |  |  |  |  |  |  |  |  |  |  |  |  |  |  |  |  |  |  |  |  |  |  |  |  |  |  |  |  |  |  |  |  |  |  |  |  |  |  |  |  |  |  |  |  |  |  |  |  |  |  |  |  |  |  |  |  |  |  |  |  |  |  |  |  |  |  |  |  |  |  |  |  |  |  |  |  |  |  |  |  |  |  |  |  |  |  |  |  |  |  |  |  |  |  |  |  |  |  |  |  |  |  |  |  |  |  |  |  |  |  |  |  |  |  |  |  |  |  |  |  |  |  |  |  |  |  |  |  |  |  |  |  |  |  |  |  |  |  |  |  |  |  |  |  |  |  |  |  |  |  |  |  |  |  |  |  |  |  |  |  |  |  |  |  |  |  |  |  |  |  |  |  |  |  |  |  |  |  |  |  |  |  |  |  |  |  |  |  |  |  |  |  |  |  |  |  |  |  |  |  |  |  |  |  |  |  |  |  |  |  |  |  |  |  |  |  |  |  |  |  |  |  |  |  |  |  |  |  |  |  |  |  |  |  |  |  |  |  |  |  |  |  |  |  |  |  |  |  |  |  |  |  |  |  |  |  |  |  |  |  |  |  |  |  |  |  |  |  |  |  |  |  |  |  |  |  |  |  |  |  |  |  |  |  |  |  |  |  |  |  |  |  |  |  |  |  |  |  |  |  |  |  |  |  |  |  |  |  |  |  |  |  |  |  |  |  |  |  |  |  |  |  |  |  |  |  |  |  |  |  |  |  |  |  |  |  |  |  |  |  |  |  |  |  |  |  |  |  |  |  |  |  |  |  |  |  |  |  |  |  |  |  |  |  |  |  |  |  |  |  |  |  |  |  |  |  |  |  |  |  |  |  |  |  |  |  |  |  |
| --- | --- | --- | --- | --- | --- | --- | --- | --- | --- | --- | --- | --- | --- | --- | --- | --- | --- | --- | --- | --- | --- | --- | --- | --- | --- | --- | --- | --- | --- | --- | --- | --- | --- | --- | --- | --- | --- | --- | --- | --- | --- | --- | --- | --- | --- | --- | --- | --- | --- | --- | --- | --- | --- | --- | --- | --- | --- | --- | --- | --- | --- | --- | --- | --- | --- | --- | --- | --- | --- | --- | --- | --- | --- | --- | --- | --- | --- | --- | --- | --- | --- | --- | --- | --- | --- | --- | --- | --- | --- | --- | --- | --- | --- | --- | --- | --- | --- | --- | --- | --- | --- | --- | --- | --- | --- | --- | --- | --- | --- | --- | --- | --- | --- | --- | --- | --- | --- | --- | --- | --- | --- | --- | --- | --- | --- | --- | --- | --- | --- | --- | --- | --- | --- | --- | --- | --- | --- | --- | --- | --- | --- | --- | --- | --- | --- | --- | --- | --- | --- | --- | --- | --- | --- | --- | --- | --- | --- | --- | --- | --- | --- | --- | --- | --- | --- | --- | --- | --- | --- | --- | --- | --- | --- | --- | --- | --- | --- | --- | --- | --- | --- | --- | --- | --- | --- | --- | --- | --- | --- | --- | --- | --- | --- | --- | --- | --- | --- | --- | --- | --- | --- | --- | --- | --- | --- | --- | --- | --- | --- | --- | --- | --- | --- | --- | --- | --- | --- | --- | --- | --- | --- | --- | --- | --- | --- | --- | --- | --- | --- | --- | --- | --- | --- | --- | --- | --- | --- | --- | --- | --- | --- | --- | --- | --- | --- | --- | --- | --- | --- | --- | --- | --- | --- | --- | --- | --- | --- | --- | --- | --- | --- | --- | --- | --- | --- | --- | --- | --- | --- | --- | --- | --- | --- | --- | --- | --- | --- | --- | --- | --- | --- | --- | --- | --- | --- | --- | --- | --- | --- | --- | --- | --- | --- | --- | --- | --- | --- | --- | --- | --- | --- | --- | --- | --- | --- | --- | --- | --- | --- | --- | --- | --- | --- | --- | --- | --- | --- | --- | --- | --- | --- | --- | --- | --- | --- | --- | --- | --- | --- | --- | --- | --- | --- | --- | --- | --- | --- | --- | --- | --- | --- | --- | --- | --- | --- | --- | --- | --- | --- | --- | --- | --- | --- | --- | --- | --- | --- | --- | --- | --- | --- | --- | --- | --- | --- | --- | --- | --- | --- | --- | --- | --- | --- | --- | --- | --- | --- | --- | --- | --- | --- | --- | --- | --- | --- | --- | --- | --- | --- | --- | --- | --- | --- | --- | --- | --- | --- | --- | --- | --- | --- | --- | --- | --- | --- | --- | --- | --- | --- | --- | --- | --- | --- | --- | --- | --- | --- | --- | --- | --- | --- | --- | --- | --- | --- | --- | --- | --- | --- | --- | --- | --- | --- | --- | --- | --- | --- | --- | --- | --- | --- | --- | --- | --- | --- | --- | --- | --- | --- | --- | --- | --- | --- | --- | --- | --- | --- | --- | --- | --- | --- | --- | --- | --- | --- | --- | --- | --- | --- | --- | --- | --- | --- | --- | --- | --- | --- | --- | --- | --- | --- | --- | --- | --- | --- | --- | --- | --- | --- | --- | --- | --- | --- | --- | --- | --- | --- | --- | --- | --- | --- | --- | --- | --- | --- | --- | --- | --- | --- | --- | --- | --- | --- | --- | --- | --- | --- | --- | --- | --- | --- | --- | --- | --- | --- | --- | --- | --- | --- | --- | --- | --- | --- | --- | --- | --- | --- | --- | --- | --- | --- | --- | --- | --- | --- | --- | --- | --- | --- | --- | --- | --- | --- | --- | --- | --- | --- | --- | --- | --- | --- | --- | --- | --- | --- | --- | --- | --- | --- | --- | --- | --- | --- | --- | --- | --- | --- | --- | --- | --- | --- | --- | --- | --- | --- | --- | --- | --- | --- | --- | --- | --- | --- | --- | --- | --- | --- | --- | --- | --- | --- | --- | --- | --- | --- | --- | --- | --- | --- | --- | --- | --- | --- | --- | --- | --- | --- | --- | --- | --- | --- | --- | --- | --- | --- | --- | --- | --- | --- | --- | --- | --- | --- | --- | --- | --- | --- | --- | --- | --- | --- | --- | --- | --- | --- | --- | --- | --- | --- | --- | --- | --- | --- | --- | --- | --- | --- | --- | --- | --- | --- | --- | --- | --- | --- | --- | --- | --- | --- | --- | --- | --- | --- | --- | --- | --- | --- | --- | --- | --- | --- | --- | --- | --- | --- | --- | --- | --- | --- | --- | --- | --- | --- | --- | --- | --- | --- | --- | --- | --- | --- | --- | --- | --- | --- | --- | --- | --- | --- | --- | --- | --- | --- | --- | --- | --- | --- | --- | --- | --- | --- | --- | --- | --- | --- | --- | --- | --- | --- | --- | --- | --- | --- | --- | --- | --- | --- | --- | --- | --- | --- | --- | --- | --- | --- | --- | --- | --- | --- | --- | --- | --- | --- | --- | --- | --- | --- | --- | --- | --- | --- | --- | --- | --- | --- | --- | --- | --- | --- | --- | --- | --- | --- | --- | --- | --- | --- | --- | --- | --- | --- | --- | --- | --- | --- | --- | --- | --- | --- | --- | --- | --- | --- | --- | --- | --- | --- | --- | --- | --- | --- | --- | --- | --- | --- | --- | --- | --- | --- | --- | --- | --- | --- | --- | --- | --- | --- | --- | --- | --- | --- | --- | --- | --- | --- | --- | --- | --- | --- | --- | --- | --- | --- | --- | --- | --- | --- | --- | --- | --- | --- | --- | --- | --- | --- | --- | --- | --- | --- | --- | --- | --- | --- | --- | --- | --- | --- | --- | --- | --- | --- | --- | --- | --- | --- | --- | --- | --- | --- | --- | --- | --- | --- | --- | --- | --- | --- | --- | --- | --- | --- | --- | --- | --- | --- | --- | --- | --- | --- | --- | --- | --- | --- | --- | --- | --- | --- | --- | --- | --- | --- | --- | --- | --- | --- | --- | --- | --- | --- | --- | --- | --- | --- | --- | --- | --- | --- | --- | --- | --- | --- | --- | --- | --- | --- | --- | --- | --- | --- | --- | --- | --- | --- | --- | --- | --- | --- | --- | --- | --- | --- | --- | --- | --- | --- | --- | --- | --- | --- | --- | --- | --- | --- | --- | --- | --- | --- | --- | --- | --- | --- | --- | --- | --- | --- | --- | --- | --- | --- | --- | --- | --- | --- | --- | --- | --- | --- | --- | --- | --- | --- | --- | --- | --- | --- | --- | --- | --- | --- | --- | --- | --- | --- | --- | --- | --- | --- | --- | --- | --- | --- | --- | --- | --- | --- | --- | --- | --- | --- | --- | --- | --- | --- | --- | --- | --- | --- | --- | --- | --- | --- | --- | --- | --- | --- | --- | --- | --- | --- | --- | --- | --- | --- | --- | --- | --- | --- | --- | --- | --- | --- | --- | --- | --- | --- | --- | --- | --- | --- | --- | --- | --- | --- | --- | --- | --- | --- | --- | --- | --- | --- | --- | --- | --- | --- | --- | --- | --- | --- | --- | --- |
| Huberarchaea cell legend (click on numbers for pathway map): **reliable+questionable |unique** | CG02\_land\_8\_20\_14\_3\_00\_150\_Huberarchaea\_01\_31\_209 | CG03\_land\_8\_20\_14\_0\_80\_cor\_Huberarchaea\_31\_114 | CG17\_big\_fil\_post\_rev\_8\_21\_14\_2\_50\_Huberarchaea\_31\_73 | CG18\_big\_fil\_WC\_8\_21\_14\_2\_50\_Huberarchaea\_31\_19 | CG1\_02\_FULL\_Huberarchaea\_31\_31\_curated | CG2\_30\_FULL\_Huberarchaea\_31\_98\_curated | CG\_4\_10\_14\_0\_8\_um\_filter\_cor\_Huberarchaea\_31\_133 | CG\_4\_8\_14\_3\_um\_filter\_Huberarchaea\_31\_151 | CG\_4\_9\_14\_0\_8\_um\_filter\_Huberarchaea\_31\_21 | CG\_4\_9\_14\_3\_um\_filter\_150\_Huberarchaea\_31\_125 | CG\_SAG\_2014\_w16S\_CG\_CPArch01\_01\_32\_1 ||  |
| **METABOLISM** |  |  |  |  |  |  |  |  |  |  |  |
| **Carbohydrate metabolism** |  |  |  |  |  |  |  |  |  |  |  |
| Pentose phosphate pathway | **1+0=1(u:1)** | **1+0=1(u:1)** | **1+0=1(u:1)** | **1+0=1(u:1)** | **1+0=1(u:1)** | **1+0=1(u:1)** | **1+0=1(u:1)** | **1+0=1(u:1)** | **1+0=1(u:1)** | **1+0=1(u:1)** | - |
| Galactose metabolism | - | 1+0=1(u:0) | - | - | - | - | - | - | - | - | - |
| Starch and sucrose metabolism | **1+0=1(u:1)** | **2+0=2(u:1)** | **1+0=1(u:1)** | **1+0=1(u:1)** | **1+0=1(u:1)** | **1+0=1(u:1)** | **1+0=1(u:1)** | **1+0=1(u:1)** | **1+0=1(u:1)** | **1+0=1(u:1)** | - |
| Amino sugar and nucleotide sugar metabolism | **1+0=1(u:1)** | **4+1=5(u:2)** | **1+0=1(u:1)** | **1+0=1(u:1)** | **1+0=1(u:1)** | **1+0=1(u:1)** | **2+0=2(u:2)** | **1+0=1(u:1)** | **1+0=1(u:1)** | **1+0=1(u:1)** | - |
| Pyruvate metabolism | 1+0=1(u:0) | 1+0=1(u:0) | 1+0=1(u:0) | 1+0=1(u:0) | 1+0=1(u:0) | 1+0=1(u:0) | 1+0=1(u:0) | 1+0=1(u:0) | 1+0=1(u:0) | 1+0=1(u:0) | - |
| **Energy metabolism** |  |  |  |  |  |  |  |  |  |  |  |
| Oxidative phosphorylation | **1+0=1(u:1)** | **1+0=1(u:1)** | **1+0=1(u:1)** | **1+0=1(u:1)** | **1+0=1(u:1)** | **1+0=1(u:1)** | **1+0=1(u:1)** | **1+0=1(u:1)** | **1+0=1(u:1)** | **1+0=1(u:1)** | - |
| Sulfur metabolism | - | - | - | - | - | - | 1+0=1(u:0) | - | 1+0=1(u:0) | - | - |
| **Lipid metabolism** |  |  |  |  |  |  |  |  |  |  |  |
| Glycerolipid metabolism | - | - | - | - | - | - | - | - | - | - | **1+0=1(u:1)** |
| Glycerophospholipid metabolism | **1+0=1(u:1)** | **1+0=1(u:1)** | **1+0=1(u:1)** | **1+0=1(u:1)** | **1+0=1(u:1)** | **1+0=1(u:1)** | **1+0=1(u:1)** | **1+0=1(u:1)** | **1+0=1(u:1)** | **1+0=1(u:1)** | **2+0=2(u:1)** |
| **Nucleotide metabolism** |  |  |  |  |  |  |  |  |  |  |  |
| Purine metabolism | 10+1=11(u:0) | 12+1=13(u:0) | 12+1=13(u:0) | 12+1=13(u:0) | 11+1=12(u:0) | 12+1=13(u:0) | 12+1=13(u:0) | 12+1=13(u:0) | 12+2=14(u:0) | 12+1=13(u:0) | 4+1=5(u:0) |
| Pyrimidine metabolism | **15+1=16(u:3)** | **17+1=18(u:3)** | **17+1=18(u:3)** | **17+1=18(u:3)** | **16+1=17(u:3)** | **15+1=16(u:2)** | **17+1=18(u:3)** | **17+1=18(u:3)** | **15+2=17(u:2)** | **17+1=18(u:3)** | 5+1=6(u:0) |
| **Amino acid metabolism** |  |  |  |  |  |  |  |  |  |  |  |
| Alanine, aspartate and glutamate metabolism | 1+1=2(u:0) | 1+1=2(u:0) | 1+1=2(u:0) | 1+1=2(u:0) | 1+1=2(u:0) | 1+1=2(u:0) | 1+1=2(u:0) | 1+1=2(u:0) | 1+1=2(u:0) | 1+1=2(u:0) | - |
| Glycine, serine and threonine metabolism | - | - | - | - | - | - | - | - | - | - | 1+0=1(u:0) |
| Cysteine and methionine metabolism | 1+0=1(u:0) | 1+0=1(u:0) | 1+0=1(u:0) | 1+0=1(u:0) | 1+0=1(u:0) | 1+0=1(u:0) | 1+0=1(u:0) | 1+0=1(u:0) | 1+0=1(u:0) | **2+0=2(u:1)** | - |
| Arginine and proline metabolism | **2+0=2(u:1)** | **2+0=2(u:1)** | **2+0=2(u:1)** | **2+0=2(u:1)** | **2+0=2(u:1)** | **2+0=2(u:1)** | **2+0=2(u:1)** | **2+0=2(u:1)** | **2+0=2(u:1)** | **2+0=2(u:1)** | - |
| Tyrosine metabolism | 1+0=1(u:0) | 1+0=1(u:0) | 1+0=1(u:0) | 1+0=1(u:0) | 1+0=1(u:0) | 1+0=1(u:0) | 1+0=1(u:0) | 1+0=1(u:0) | 1+0=1(u:0) | 1+0=1(u:0) | - |
| Phenylalanine metabolism | 1+0=1(u:0) | 1+0=1(u:0) | 1+0=1(u:0) | 1+0=1(u:0) | 1+0=1(u:0) | 1+0=1(u:0) | 1+0=1(u:0) | 1+0=1(u:0) | 1+0=1(u:0) | 1+0=1(u:0) | - |
| Phenylalanine, tyrosine and tryptophan biosynthesis | 2+0=2(u:0) | 2+0=2(u:0) | 2+0=2(u:0) | 2+0=2(u:0) | 2+0=2(u:0) | 2+0=2(u:0) | 2+0=2(u:0) | 2+0=2(u:0) | 2+0=2(u:0) | 2+0=2(u:0) | - |
| **Metabolism of other amino acids** |  |  |  |  |  |  |  |  |  |  |  |
| Selenocompound metabolism | 2+0=2(u:0) | 2+1=3(u:0) | 2+0=2(u:0) | 2+1=3(u:0) | 2+1=3(u:0) | 2+0=2(u:0) | 2+1=3(u:0) | 2+0=2(u:0) | 2+1=3(u:0) | 2+0=2(u:0) | 2+0=2(u:0) |
| **Glycan biosynthesis and metabolism** |  |  |  |  |  |  |  |  |  |  |  |
| N-Glycan biosynthesis | **3+0=3(u:2)** | **3+0=3(u:2)** | **3+0=3(u:2)** | **3+1=4(u:2)** | **3+0=3(u:2)** | **3+1=4(u:2)** | **2+1=3(u:2)** | **3+0=3(u:2)** | **3+0=3(u:2)** | **3+0=3(u:2)** | **1+0=1(u:1)** |
| Various types of N-glycan biosynthesis | 1+0=1(u:0) | 1+0=1(u:0) | 1+0=1(u:0) | 1+0=1(u:0) | 1+0=1(u:0) | 1+0=1(u:0) | - | 1+0=1(u:0) | 1+0=1(u:0) | 1+0=1(u:0) | - |
| **Metabolism of cofactors and vitamins** |  |  |  |  |  |  |  |  |  |  |  |
| One carbon pool by folate | 1+0=1(u:0) | 1+0=1(u:0) | 1+0=1(u:0) | 1+0=1(u:0) | 1+0=1(u:0) | - | 1+0=1(u:0) | 1+0=1(u:0) | - | 1+0=1(u:0) | - |
| Thiamine metabolism | 1+0=1(u:0) | 2+0=2(u:0) | 2+0=2(u:0) | 2+0=2(u:0) | 1+0=1(u:0) | - | 2+0=2(u:0) | 2+0=2(u:0) | 1+0=1(u:0) | 1+0=1(u:0) | - |
| Riboflavin metabolism | - | - | - | - | - | - | 1+0=1(u:0) | - | 1+0=1(u:0) | - | - |
| Porphyrin and chlorophyll metabolism | 1+1=2(u:0) | 1+1=2(u:0) | 1+1=2(u:0) | 1+1=2(u:0) | 1+1=2(u:0) | 1+1=2(u:0) | 1+1=2(u:0) | 1+1=2(u:0) | 1+1=2(u:0) | 1+1=2(u:0) | 1+0=1(u:0) |
| **Metabolism of terpenoids and polyketides** |  |  |  |  |  |  |  |  |  |  |  |
| Polyketide sugar unit biosynthesis | - | **6+0=6(u:2)** | - | 1+0=1(u:0) | 1+0=1(u:0) | - | **5+0=5(u:1)** | 4+0=4(u:0) | 1+0=1(u:0) | - | - |
| Terpenoid backbone biosynthesis | **2+0=2(u:2)** | **2+0=2(u:2)** | **2+0=2(u:2)** | **2+0=2(u:2)** | **1+0=1(u:1)** | **1+0=1(u:1)** | **2+0=2(u:2)** | **2+0=2(u:2)** | **1+0=1(u:1)** | **2+0=2(u:2)** | - |
| Biosynthesis of vancomycin group antibiotics | - | 1+0=1(u:0) | - | - | - | - | 1+0=1(u:0) | 1+0=1(u:0) | - | - | - |
| **Biosynthesis of other secondary metabolites** |  |  |  |  |  |  |  |  |  |  |  |
| Penicillin and cephalosporin biosynthesis | - | **1+0=1(u:1)** | - | - | - | - | - | - | - | - | - |
| Novobiocin biosynthesis | 2+0=2(u:0) | 2+0=2(u:0) | 2+0=2(u:0) | 2+0=2(u:0) | 2+0=2(u:0) | 2+0=2(u:0) | 2+0=2(u:0) | 2+0=2(u:0) | 2+0=2(u:0) | 2+0=2(u:0) | - |
| Streptomycin biosynthesis | - | 4+0=4(u:0) | - | 1+0=1(u:0) | 1+0=1(u:0) | - | 4+0=4(u:0) | 4+0=4(u:0) | 1+0=1(u:0) | - | - |
| Isoquinoline alkaloid biosynthesis | 1+0=1(u:0) | 1+0=1(u:0) | 1+0=1(u:0) | 1+0=1(u:0) | 1+0=1(u:0) | 1+0=1(u:0) | 1+0=1(u:0) | 1+0=1(u:0) | 1+0=1(u:0) | 1+0=1(u:0) | - |
| Tropane, piperidine and pyridine alkaloid biosynthesis | 1+0=1(u:0) | 1+0=1(u:0) | 1+0=1(u:0) | 1+0=1(u:0) | 1+0=1(u:0) | 1+0=1(u:0) | 1+0=1(u:0) | 1+0=1(u:0) | 1+0=1(u:0) | 1+0=1(u:0) | - |
|  |
| **GENETIC INFORMATION PROCESSING** |  |  |  |  |  |  |  |  |  |  |  |
| **Transcription** |  |  |  |  |  |  |  |  |  |  |  |
| RNA polymerase | 6+1=7(u:0) | 7+1=8(u:0) | 7+1=8(u:0) | 7+1=8(u:0) | 6+1=7(u:0) | 7+1=8(u:0) | 7+1=8(u:0) | 7+1=8(u:0) | 7+2=9(u:0) | 7+1=8(u:0) | 2+1=3(u:0) |
| Basal transcription factors | **3+0=3(u:2)** | **3+1=4(u:2)** | **3+0=3(u:2)** | **3+0=3(u:2)** | **2+0=2(u:1)** | **3+0=3(u:2)** | **3+1=4(u:2)** | **3+0=3(u:2)** | **3+0=3(u:2)** | **3+1=4(u:2)** | - |
| Spliceosome | 1+0=1(u:0) | 1+0=1(u:0) | 1+0=1(u:0) | 1+0=1(u:0) | 1+0=1(u:0) | 1+0=1(u:0) | 1+0=1(u:0) | 1+0=1(u:0) | 1+0=1(u:0) | 1+0=1(u:0) | - |
| **Translation** |  |  |  |  |  |  |  |  |  |  |  |
| Aminoacyl-tRNA biosynthesis | **23+0=23(u:21)** | **23+0=23(u:21)** | **23+0=23(u:21)** | **23+0=23(u:21)** | **22+0=22(u:20)** | **20+0=20(u:18)** | **23+0=23(u:21)** | **23+0=23(u:21)** | **23+0=23(u:21)** | **23+0=23(u:21)** | **10+0=10(u:8)** |
| Ribosome biogenesis in eukaryotes | **8+0=8(u:4)** | **8+0=8(u:4)** | **8+0=8(u:4)** | **8+0=8(u:4)** | **6+0=6(u:2)** | **7+0=7(u:3)** | **7+0=7(u:4)** | **8+0=8(u:4)** | **8+0=8(u:4)** | **8+0=8(u:4)** | **4+0=4(u:2)** |
| Ribosome | **33+12=45(u:32)** | **36+11=47(u:35)** | **33+10=43(u:32)** | **35+11=46(u:34)** | **34+10=44(u:33)** | **32+10=42(u:31)** | **36+11=47(u:35)** | **33+11=44(u:32)** | **36+11=47(u:35)** | **34+11=45(u:33)** | **11+3=14(u:11)** |
| RNA transport | **9+0=9(u:6)** | **10+0=10(u:7)** | **10+0=10(u:7)** | **9+0=9(u:6)** | **9+0=9(u:6)** | **9+0=9(u:6)** | **9+0=9(u:7)** | **10+0=10(u:7)** | **10+0=10(u:7)** | **10+0=10(u:7)** | **3+0=3(u:1)** |
| mRNA surveillance pathway | **3+0=3(u:3)** | **3+0=3(u:3)** | **3+0=3(u:3)** | **3+0=3(u:3)** | **2+0=2(u:2)** | **3+0=3(u:3)** | **3+0=3(u:3)** | **3+0=3(u:3)** | **3+0=3(u:3)** | **3+0=3(u:3)** | **1+0=1(u:1)** |
| **Folding, sorting and degradation** |  |  |  |  |  |  |  |  |  |  |  |
| RNA degradation | **3+0=3(u:3)** | **4+0=4(u:4)** | **3+0=3(u:3)** | **3+0=3(u:3)** | **3+0=3(u:3)** | **3+0=3(u:3)** | **4+0=4(u:4)** | **3+0=3(u:3)** | **3+0=3(u:3)** | **3+0=3(u:3)** | **1+0=1(u:1)** |
| Proteasome | **2+1=3(u:2)** | **2+1=3(u:2)** | **2+1=3(u:2)** | **2+1=3(u:2)** | **2+1=3(u:2)** | **2+1=3(u:2)** | **2+1=3(u:2)** | **2+1=3(u:2)** | **2+1=3(u:2)** | **1+1=2(u:1)** | **1+0=1(u:1)** |
| Protein export | **5+2=7(u:1)** | **5+2=7(u:1)** | **5+2=7(u:1)** | **5+2=7(u:1)** | **5+2=7(u:1)** | **5+2=7(u:1)** | **5+2=7(u:1)** | **5+2=7(u:1)** | **5+2=7(u:1)** | **5+2=7(u:1)** | 1+0=1(u:0) |
| Ubiquitin mediated proteolysis | - | - | - | - | - | - | 1+0=1(u:0) | - | - | - | - |
| Sulfur relay system | 1+0=1(u:0) | 2+0=2(u:0) | 2+0=2(u:0) | 2+0=2(u:0) | 1+0=1(u:0) | - | 2+0=2(u:0) | 2+0=2(u:0) | 1+0=1(u:0) | 1+0=1(u:0) | - |
| Protein processing in endoplasmic reticulum | **5+1=6(u:3)** | **4+1=5(u:2)** | **4+1=5(u:2)** | **4+1=5(u:2)** | **4+1=5(u:2)** | **4+1=5(u:2)** | **4+2=6(u:3)** | **4+1=5(u:2)** | **4+1=5(u:2)** | **4+0=4(u:2)** | **3+0=3(u:2)** |
| **Replication and repair** |  |  |  |  |  |  |  |  |  |  |  |
| DNA replication | **11+0=11(u:5)** | **13+0=13(u:6)** | **12+0=12(u:5)** | **12+0=12(u:5)** | **12+0=12(u:5)** | **12+0=12(u:5)** | **13+0=13(u:6)** | **12+0=12(u:5)** | **12+0=12(u:5)** | **10+1=11(u:4)** | **6+0=6(u:3)** |
| Base excision repair | **5+0=5(u:2)** | **5+0=5(u:2)** | **5+0=5(u:2)** | **5+0=5(u:2)** | **5+0=5(u:2)** | **5+0=5(u:2)** | **5+0=5(u:2)** | **4+0=4(u:1)** | **5+0=5(u:2)** | **5+0=5(u:2)** | 2+0=2(u:0) |
| Nucleotide excision repair | 5+0=5(u:0) | 5+0=5(u:0) | 5+0=5(u:0) | 5+0=5(u:0) | 5+0=5(u:0) | 5+0=5(u:0) | 5+0=5(u:0) | 5+0=5(u:0) | 5+0=5(u:0) | 4+0=4(u:0) | 2+0=2(u:0) |
| Mismatch repair | **5+0=5(u:1)** | **5+0=5(u:1)** | **5+0=5(u:1)** | **5+0=5(u:1)** | **5+0=5(u:1)** | **5+0=5(u:1)** | 4+0=4(u:0) | 4+0=4(u:0) | **5+0=5(u:1)** | **4+0=4(u:1)** | 2+0=2(u:0) |
| Homologous recombination | 1+0=1(u:0) | 1+0=1(u:0) | 1+0=1(u:0) | 1+0=1(u:0) | 1+0=1(u:0) | 1+0=1(u:0) | 1+0=1(u:0) | 1+0=1(u:0) | 1+0=1(u:0) | - | - |
| Non-homologous end-joining | 1+0=1(u:0) | 1+0=1(u:0) | 1+0=1(u:0) | 1+0=1(u:0) | 1+0=1(u:0) | 1+0=1(u:0) | 1+0=1(u:0) | 1+0=1(u:0) | 1+0=1(u:0) | 1+0=1(u:0) | - |
| Fanconi anemia pathway | **2+0=2(u:1)** | **2+0=2(u:1)** | **2+0=2(u:1)** | **2+0=2(u:1)** | **2+0=2(u:1)** | **2+0=2(u:1)** | **2+0=2(u:1)** | **2+0=2(u:1)** | **2+0=2(u:1)** | **1+0=1(u:1)** | **1+0=1(u:1)** |
|  |
| **ENVIRONMENTAL INFORMATION PROCESSING** |  |  |  |  |  |  |  |  |  |  |  |
| **Membrane transport** |  |  |  |  |  |  |  |  |  |  |  |
| Bacterial secretion system | **4+2=6(u:1)** | **4+2=6(u:1)** | **4+2=6(u:1)** | **4+2=6(u:1)** | **4+2=6(u:1)** | **4+2=6(u:1)** | **4+2=6(u:1)** | **4+2=6(u:1)** | **4+2=6(u:1)** | **4+2=6(u:1)** | **1+0=1(u:1)** |
| **Signal transduction** |  |  |  |  |  |  |  |  |  |  |  |
| Two-component system | **2+0=2(u:1)** | **2+0=2(u:1)** | **2+0=2(u:1)** | **2+0=2(u:1)** | **2+0=2(u:1)** | **2+0=2(u:1)** | **2+0=2(u:1)** | **2+0=2(u:1)** | **2+0=2(u:1)** | **2+0=2(u:1)** | - |
| HIF-1 signaling pathway | 1+0=1(u:0) | 1+0=1(u:0) | 1+0=1(u:0) | 1+0=1(u:0) | 1+0=1(u:0) | 1+0=1(u:0) | 1+0=1(u:0) | 1+0=1(u:0) | 1+0=1(u:0) | 1+0=1(u:0) | - |
| mTOR signaling pathway | 1+0=1(u:0) | 1+0=1(u:0) | 1+0=1(u:0) | 1+0=1(u:0) | 1+0=1(u:0) | 1+0=1(u:0) | 1+0=1(u:0) | 1+0=1(u:0) | 1+0=1(u:0) | 1+0=1(u:0) | - |
| PI3K-Akt signaling pathway | 1+0=1(u:0) | 1+0=1(u:0) | 1+0=1(u:0) | 1+0=1(u:0) | 1+0=1(u:0) | 1+0=1(u:0) | 1+0=1(u:0) | 1+0=1(u:0) | 1+0=1(u:0) | 1+0=1(u:0) | - |
| AMPK signaling pathway | **1+0=1(u:1)** | **1+0=1(u:1)** | **1+0=1(u:1)** | **1+0=1(u:1)** | **1+0=1(u:1)** | **1+0=1(u:1)** | **1+0=1(u:1)** | **1+0=1(u:1)** | **1+0=1(u:1)** | **1+0=1(u:1)** | - |
|  |
| **CELLULAR PROCESSES** |  |  |  |  |  |  |  |  |  |  |  |
| **Transport and catabolism** |  |  |  |  |  |  |  |  |  |  |  |
| Phagosome | 1+0=1(u:0) | 1+0=1(u:0) | 1+0=1(u:0) | 1+0=1(u:0) | 1+0=1(u:0) | 1+0=1(u:0) | 1+0=1(u:0) | 1+0=1(u:0) | 1+0=1(u:0) | 1+0=1(u:0) | 1+0=1(u:0) |
| **Cell growth and death** |  |  |  |  |  |  |  |  |  |  |  |
| Cell cycle | 1+0=1(u:0) | 1+0=1(u:0) | 1+0=1(u:0) | 1+0=1(u:0) | 1+0=1(u:0) | 1+0=1(u:0) | 2+0=2(u:0) | 1+0=1(u:0) | 1+0=1(u:0) | 1+0=1(u:0) | 1+0=1(u:0) |
| Cell cycle - yeast | - | - | - | - | - | - | 1+0=1(u:0) | - | - | - | - |
| Cell cycle - Caulobacter | **1+0=1(u:1)** | **1+0=1(u:1)** | **1+0=1(u:1)** | **1+0=1(u:1)** | **1+0=1(u:1)** | **1+0=1(u:1)** | **1+0=1(u:1)** | **1+1=2(u:1)** | **1+0=1(u:1)** | **1+0=1(u:1)** | **2+0=2(u:2)** |
| Meiosis - yeast | **1+0=1(u:1)** | **1+0=1(u:1)** | **1+0=1(u:1)** | **1+0=1(u:1)** | **1+0=1(u:1)** | **1+0=1(u:1)** | **2+0=2(u:1)** | **1+0=1(u:1)** | **1+0=1(u:1)** | **1+0=1(u:1)** | - |
| Oocyte meiosis | - | - | - | - | - | - | 1+0=1(u:0) | - | - | - | - |
